# Dengue Virus Replication Is Associated with Catecholamine Biosynthesis and Metabolism in Hepatocytes

**DOI:** 10.3390/v14030564

**Published:** 2022-03-09

**Authors:** George Mpekoulis, Vassilina Tsopela, Anna Chalari, Katerina I. Kalliampakou, Georgios Panos, Efseveia Frakolaki, Raphaela S. Milona, Diamantis C. Sideris, Dido Vassilacopoulou, Niki Vassilaki

**Affiliations:** 1 Laboratory of Molecular Virology, Hellenic Pasteur Institute, 11521 Athens, Greece; g.mpekoulis@pasteur.gr (G.M.); vas.tsopela@gmail.com (V.T.); annach_91@yahoo.gr (A.C.); kalliamp@yahoo.gr (K.I.K.); panos.georgios.bio@gmail.com (G.P.); raphaelasmilona@gmail.com (R.S.M.); 2 Section of Biochemistry and Molecular Biology, Faculty of Biology, National and Kapodistrian University of Athens, 15701 Athens, Greece; dsideris@biol.uoa.gr (D.C.S.); didovass@biol.uoa.gr (D.V.)

**Keywords:** dengue virus replication, dopamine and norepinephrine biosynthesis, L-Dopa decarboxylase, dopamine beta-hydroxylase, catecholamine metabolism, vesicular monoamine transporter 2

## Abstract

Previously, the association between the catecholamine biosynthetic enzyme L-Dopa decarboxylase (DDC) and Dengue virus (DV) replication was demonstrated in liver cells and was found to be mediated at least by the interaction between DDC and phosphoinositide 3-kinase (PI3K). Here, we show that biogenic amines production and uptake impede DV replication in hepatocytes and monocytes, while the virus reduces catecholamine biosynthesis, metabolism, and transport. To examine how catecholamine biosynthesis/metabolism influences DV, first, we verified the role of DDC by altering DDC expression. DDC silencing enhanced virus replication, but not translation, attenuated the negative effect of DDC substrates on the virus and reduced the infection related cell death. Then, the role of the downstream steps of the catecholamine biosynthesis/metabolism was analyzed by chemical inhibition of the respective enzymes, application of their substrates and/or their products; moreover, reserpine, the inhibitor of the vesicular monoamine transporter 2 (VMAT2), was used to examine the role of uptake/storage of catecholamines on DV. Apart from the role of each enzyme/transporter, these studies revealed that the dopamine uptake, and not the dopamine-signaling, is responsible for the negative effect on DV. Accordingly, all treatments expected to enhance the accumulation of catecholamines in the cell cytosol suppressed DV replication. This was verified by the use of chemical inducers of catecholamine biosynthesis. Last, the cellular redox alterations due to catecholamine oxidation were not related with the inhibition of DV replication. In turn, DV apart from its negative impact on DDC, inhibits tyrosine hydroxylase, dopamine beta-hydroxylase, monoamine oxidase, and VMAT2 expression.

## 1. Introduction

Dengue virus (DV) poses a considerable public health problem in over 100 countries, with a high possibility of further transmission [1]. Infection with any of the four DV serotypes may result in a wide spectrum of clinical symptoms ranging from mild dengue fever (DF) to the more severe dengue hemorrhagic fever (DHF) and the life-threatening dengue shock syndrome (DSS) [2]. High viremia levels in DV infection have been associated with the involvement of different organs, such as the liver and brain, in the severe form of the disease [3]. Especially, the liver is the most commonly involved organ in dengue. The only approved dengue vaccine has limited efficacy and safety issues [4], while an effective therapeutic agent for treating DV infection is unavailable to date [5]. DV is an enveloped virus possessing a positive sense, single-stranded m7G-capped RNA genome, which encodes a single polyprotein [6,7]. This is proteolytically cleaved into structural proteins (C, prM, and E), that are involved in receptor binding, virus fusion, and virion assembly, and non-structural (NS) proteins (NS1, NS2A, NS2B, NS3, NS4A, NS4B, and NS5), that are responsible for the replication of the viral genome and critical for the evasion from host cell immune responses. In vitro, viral replication can occur in cells of a broad range of tissues, including hepatocytes [8,9,10].

Previous studies have highlighted that the liver is the primary place of the detoxification and metabolism of the blood circulating catecholamines [11]. Furthermore, several reports have supported that the biosynthesis of catecholamines is also active in hepatocytes, as shown by the expression of the related enzymes [12,13,14,15,16,17,18,19,20].

In this context, our previous research has revealed a bidirectional relationship between L-Dopa decarboxylase (DDC), an essential enzyme in the biosynthesis of catecholamines, and DV replication in cultured hepatocytes [21]. DDC, specifically, is a pyridoxal-5′-phosphate (PLP)-dependent enzyme that converts L-3,4-dihydroxyphenylalanine (L-Dopa) to the catecholamine dopamine (DA) and L-5-hydroxytryptophan (5-HTP) to the indolamine serotonin (5-HT) [22]. In detail, we showed that viral replication downregulates DDC mRNA and protein levels in Huh7 cells, at least through a phosphoinositide 3-kinase (PI3K)-dependent mechanism [21]. Apart from its importance in neurotransmitter biosynthesis, DDC has, also, been isolated from the liver and a variety of other organs in the periphery [23] having additional physiological role, in cell proliferation and apoptosis [24,25]. The latter has been recently suggested to be mediated by the physical and functional interaction between DDC and PI3K that is inhibitory for the kinase [25].

The pathway of biosynthesis and metabolism of catecholamines starts from tyrosine that is converted to L-Dopa by the activity of tyrosine hydroxylase (TH), while the downstream action of DDC produces DA. VMAT1 and VMAT2, two vesicular monoamine transporters, actively transfer DA and 5-HT from the cytosol into vesicles, utilizing a proton gradient generated by the vesicle membrane-localized V-ATPases to power monoamine import. The vesicle imported DA can be stored or converted to norepinephrine (NE) by the activity of the vesicle membrane-localized dopamine-beta-hydroxylase (DBH). Fusion of the monoamine-carrying vesicles with the cytoplasmic membrane allows their content to be released outside the cell. Specific transporters on the cell membrane uptake catecholamines, and VMATs appear to have an important role in this process [26,27,28,29]. In the cytosol, NE is converted to epinephrine (EPI) by norepinephrine (phenylethanolamine) N-methyltransferase (PNMT). At the physiological pH of the cytosol, catecholamines and serotonin spontaneously loose protons and autooxidate to reactive and unstable quinones. Autooxidation can be prevented by the actions of monoamino oxidases (MAO-A and MAO-B) [12,30,31]. MAOs are flavin-containing enzymes of the outer mitochondrial membrane that catalyze the oxidative deamination of catecholamines, resulting in the modulation of their concentrations. The vesicle-stored-catecholamines are protected from oxidation due to the acidic pH [32].

Limited data exist linking viral infections with monoamine biosynthesis and metabolism apart from our previously reported findings on the association of DDC with the infections by HCV and DV [21,33] and SARS-CoV-2 [34]. Increased transcription of *MAO-B* upon infection by Simian immunodeficiency virus [35] and stimulation of 5-HT release from the host cells by Rotavirus [36] and DV [37] have been shown; moreover, elevation of NE and EPI plasma levels in patients with neurological complications after Enterovirus 71 (EV71) infection with a parallel enhancement of virus titers and infectivity in human leukocyte cell lines by the two catecholamines [38] has been observed. Coxsackie type B4 virus or yellow fever virus infection in newborn mice abrogated catecholamine biosynthesis in the brain [39]; moreover, a facilitation of viral entry by dopamine D2 or D4 receptor has been shown for DV [40,41] and by 5-HT receptor for HCV [42], Reovirus [43] and JC Polyomavirus [44].

The interaction of DV virus with elements of the monoamine biosynthetic and metabolic pathway, apart from DDC, has not yet been reported. In this research, we investigated the association of DV replication with other parts of the catecholamine and serotonin biosynthetic/metabolic pathway in hepatoma cells. Except for the already suggested interaction between the DDC-PI3K complex and the DV life cycle in hepatocytes, we addressed the importance of the biosynthetic function of DDC, in addition to other proteins of the catecholamine and serotonin pathway, for viral infection; for this, we either silenced the expression or chemically inhibited/induced the related proteins, such as biosynthetic, metabolic enzymes and transporters. We also provided externally the substrates and products of the catecholamine pathway to the cells; moreover, we studied the effect of DV infection on the expression of the biosynthetic, metabolic enzymes and transporters of the biogenic amine pathway.

## 2. Materials and Methods

### 2.1. Cells

Huh7 cells [45] (kindly provided by R. Bartenschlager, University of Heidelberg, Heidelberg, Germany), VeroE6 cells (originally obtained from ATCC#CRL-1586), immortalized Human Hepatocytes (IHH) (originally obtained from ATCC) [46], THP-1 hematopoietic lineage cell line (monocytic cells) (kindly provided by E. Meurs, Institute Pasteur, Paris, France) [47] and Huh7-D2 stable cell line that harbors the DV bicistronic subgenomic replicon were used in this study. The stably expressed DV replicon has been constructed by replacing the structural protein-coding region downstream of the 5′ cyclization sequence (CS) and specifically between capsid protein codon 28 and the last 26 codons at the carboxy-terminus end of the envelope protein of the DV-2 16681 strain, with the humanized Renilla luciferase-ubiquitin-puromycin acetyltransferase (hRUPac) cassette to generate pD2-hRUPac [48,49,50] (kindly provided by C. M. Rice, The Rockefeller University, New York, NY, USA). High glucose (25 mM) Dulbecco’s modified minimal essential medium (Thermo Fisher Scientific, Waltham, MA, USA), supplemented with L-glutamine (2 mM), non-essential amino acids (0.1 mM), penicillin (100 U/mL), streptomycin (100 µg/mL), and fetal calf serum (10% *v*/*v*) (hereinafter referred to referred to as complete DMEM), was used for cell culture. Complete DMEM was supplemented with 0.25 μg/mL puromycin for Huh7-D2.

### 2.2. Viruses and Plasmid Vectors

Plasmid vectors containing the full-length dengue virus genomic sequence pFK-DVs and pFK-DVR2A (with the reporter gene Renilla luciferase inserted downstream of a duplicated sequence corresponding to the first 103 nucleotides of the DV capsid region) and the subgenomic replicon vectors pFK-sgDVR2A and pFK-sgDVR2A-GND (replication deficient NS5 mutant), derived from the 16681 strain of DV-2 have been described elsewhere [51,52]. To silence *DDC*, we used the psi-LVRH1GP/shDDC (shDDC) vector, encoding a short hairpin RNA (shRNA) that targets DDC mRNA (5′-GCTCCTTTGACAATCTCTTAG-3′). shDDC vector and the negative control vector (shControl), encoding a scramble shRNA (5′-GCTTCGCGCCGTAGTCTTA-3′), were purchased from GeneCopoeia (Rockville, MD, USA). The mammalian vector pcDNA 3.1(+)-DDC expresses the full-length DDC and has been previously described [53,54].

### 2.3. Generation of Stable Huh7.5 Derived Cell Lines Expressing shDDC or shControl RNA

Lentiviruses were produced by transient three-vector transfection of 293T cells. Briefly, the vesicular stomatitis virus envelope glycoprotein expression vector pczVSV-G [55], the HIV-1 Gag-Pol expression vector pCMV-ΔR8.74 [56] and the vector psi-LVRH1GP/shDDC (shDDC) or the respective negative control vector (shControl) were transfected using Lipofectamine 2000 (Invitrogen, Carlsbad, CA, USA) into 293T cells at a ratio of 1:3:3 (2.14 μg:6.42 μg:6.42 μg), respectively, according to the manufacturer’s instructions. Cells were previously seeded at a density of 0.5 × 10^6^ cells/well in a 6 cm diameter plate and further cultured for 24 h. The transfection medium was replaced after 8 h with fresh antibiotic-free DMEM. Supernatants containing the lentiviral pseudoparticles were harvested 48 h later, cleared by passage through 0.45 μm pore sized filters and used to inoculate 4 × 10^4^ Huh7.5 cells. 6 h later, supernatants were replaced with complete DMEM and transduced cells were selected by using medium supplemented with 2 μg/mL puromycin at 48 h post-virus inoculation and afterward. Detection of DDC mRNA and protein was used to verify *DDC* silencing.

### 2.4. In Vitro Transcription

For in vitro transcription, 10 μg DNA of the respective plasmid vector containing the dengue virus genomic sequence were linearized by digestion for 2 h with XbαΙ, extracted with phenol and chloroform, precipitated with ethanol, and dissolved in RNase-free water. In vitro transcription reaction mixtures (100 μL) contained 0.1 μg DNA/μL, 20 μL 5× SP6 reaction buffer, 12.5 μL rNTP mix (25 mM each ATP, CTP, UTP and 12.5 mM GTP) 20 μL m 7G(5′)ppp(5′)G RNA cap structure analog (5 mM), 2.5 μL RNasin (40 U/μL), and 4 μL SP6 RNA polymerase (20 U/μL). After incubation for 2.5 h at 40 °C, 20 U of SP6 RNA polymerase/μL reaction mixture was added, followed by another 2.5 h of incubation at 40 °C. Transcription was terminated by addition of 1.U of RNase-free DNase (Promega) per μg of DNA and 1h of incubation at 37 °C. The RNA extraction was performed with acidic phenol and chloroform, precipitated with isopropanol, and dissolved in RNase-free water. Denaturing agarose gel electrophoresis was used to test RNA integrity, while the concentration’s determination was identified by measuring the optical density at 260 nm.

### 2.5. Transfection Assays

Full-length DV RNAs, synthesized after in vitro transcription, were transfected to VeroE6 cells via electroporation, as mentioned elsewhere [57]. The transfection of subgenomic DV RNA, of shDDC or shControl vectors, and of pcDNA3.1(+)-DDC vector into Huh7 cells, was performed in likewise manner. Cells transfected via electroporation with a capped and polyadenylated RNA encoding the reporter Renilla luciferase, produced as previously reported [58], or with pcDNA 3.1(+) (Invitrogen), were used as negative controls in the respective experiments. In brief, resuspension (4 × 10^6^) cells were prepared in Cytomix [59] containing ATP (2 mM) and glutathione (5 mM) and mixed with 10 µg of viral RNA or 20 µg of DNA. The electroporation was done with a Gene Pulser system (Bio-Rad, Hercules, CA, USA) and cells were seeded in complete DMEM as the assay requires.

Transfection of Huh7.5 cells stably expressing shDDC or shControl was performed with in vitro produced RNAs from the subgenomic reporter vectors pFK-sgDVR2A or pFK-sgDVR2A-GND (0.5 μg RNA/0.7 × 10^4^ cells) using Lipofectamine 2000 (Invitrogen, Carlsbad, CA, USA) according to the manufacturer’s instructions.

### 2.6. Production of Virus Stocks and Infection Assays

VeroE6 cells were electroporated with 10 μg of the respective in vitro transcribed DV RNA, as described elsewhere [51]. The electroporated cells were seeded at two 10 cm plates and 24 h after transfection, the cell culture medium was replaced with DMEM containing 15 mM HEPES (pH 7.5) and the cells were further cultured until a visible cytopathic effect (CPE), characteristic of advanced DV infection was observed. Virus-containing cell culture supernatants were harvested 4 and 7 days after transfection, cleared from cell debris by filtration through a 0.45 μM syringe-tip filter, aliquoted and frozen at −80 °C. VeroE6-derived virus was used to infect VeroE6 cells seeded at four 10 cm plates. The cell culture medium was replaced with DMEM containing 15 mM HEPES (pH 7.5), 4 h post-infection. The virus-containing cell culture supernatants were harvested at 4, 5, 6 and 7 days. Virus stocks were filtered (0.45 μM filters), aliquoted and stored at −80 °C until further use.

DV virus stocks were used to infect naive Huh7 cells or Huh7.5 cells stably expressing shDDC or shControl, IHH and THP-1 cells (DOI hypoxia cells 2018). The culture medium was replaced 4 h post-virus inoculation and the cells were further cultured for the indicated time points.

### 2.7. Determination of Virus Titers

DV virus concentration was quantified by standard plaque assay (PFU) titration method on VeroE6 cells as reported previously [60]. Briefly, VeroE6 at a seeding density of 2 × 10^5^ cells/well were cultured overnight and inoculated with virus stocks at 10-fold serial dilutions for 1 h. After inoculum removal, an immobilizing overlay containing 1.5% carboxymethylcellulose (Sigma-Aldrich, Taufkirchen, Germany) in minimal essential medium (MEM) (Thermo Fisher Scientific, Waltham, MA, USA) was added to the plates. The overlaid plates remained in the incubator for seven days and subsequently 10% formaldehyde solution was applied in order to fix the cells. For visualizing plaques, cell staining was performed using 1% crystal violet (Sigma-Aldrich, Taufkirchen, Germany) in 10% methanol (20 min). Infectious virus titers were measured in plaque-forming units (PFU) per mL.

### 2.8. Gel Electrophoresis and Western Blot Analysis

Protein lysates were subjected to denaturing SDS-PAGE and immunoblotting, as reported elsewhere [61]. For immunoblotting, the following antibodies with their respective dilutions were used: DV NS3 monoclonal antibody (GeneTex International Corporation, Hsinchu City, Taiwan) at 1:4000, DDC mouse monoclonal antibody (clone 8E8; Santa Cruz Biotechnology, Dallas, TX, USA) at 1:500, VMAT2 mouse monoclonal antibody (clone H-12; Santa Cruz Biotechnology) at 1:1000, TH mouse monoclonal antibody (clone F-11; Santa Cruz Biotechnology) at 1:1000, DBH mouse monoclonal antibody (clone A-9; Santa Cruz Biotechnology) at 1:200, MAO-B mouse monoclonal antibody (clone D-6; Santa Cruz Biotechnology) at 1:500 and pan-actin mouse monoclonal antibody (Merck-Millipore, Burlington, MA, USA) at 1:6000. The secondary anti-mouse and anti-rabbit horseradish peroxidase-conjugated antibodies (Cell Signalling, Leiden, The Netherlands) were used at a concentration of 1:2000.

### 2.9. Luciferase Assay

Enzymatic activity of Renilla luciferase (RLuc) in cell lysates was assayed using 12 µM coelenterazine (Promega Corporation, Madison, WI, USA) in a reaction buffer (50 mM potassium phosphate of pH 7.4, 500 mM NaCl, 1 mM EDTA). Sample measurements were carried out in a GloMax 20/20 single-tube luminometer (Promega Corporation, Madison, WI, USA) for 10 s. Total protein concentration, determined with the Bradford protein assay (Bio-Rad, Hercules, CA, USA), was used for the normalization of luciferase activities.

### 2.10. Measurement of Cellular ATP Content

Intracellular ATP content was estimated using the ViaLight HS BioAssay kit (Lonza, Basel, Switzerland) based on the protocol provided by the manufacturer, in a GloMax 20/20 single-tube luminometer (Promega Corporation, Madison, WI, USA) for 1 s. Intracellular levels of ATP were normalized in respect to the total amount of protein.

### 2.11. RNA Quantification by Reverse Transcription-Quantitative PCR (RT-qPCR)

Total RNA extraction from cells was conducted using NucleoZOL (Macherey-Nagel, Düren, Germany) according to the manufacturer’s instructions. For cDNA synthesis, Moloney murine leukemia virus reverse transcriptase (Promega Corporation, Madison, WI, USA) was used in a protocol supplemented by the manufacturer. For the DV plus-strand RNA quantitation, reverse transcription (RT) was performed using the DV specific primer DV-A and the housekeeping gene primer YWHAZ-R (Table 1), specific for the 14-3-3-zeta polypeptide (YWHAZ), the expression of which was used to control for differences in input RNA (3.5 pmol/μL final concentration of each primer). To quantify cellular mRNAs, reverse transcription was performed using oligo(dT) primers (New England Biolabs, Ipswich, MA, USA). Real-time quantitative PCR was performed using Luna^®^ Universal qPCR Master Mix (New England Biolabs, Inc. Ipswich, MA, USA) as well as primer pairs specific for the DV IRES (DV-A and DV-S), the exons 10-12 of full-length DDC mRNA, TH, DBH, MAO-A, MAO-B, VMAT2, OCT1 and HO-1 mRNAs. YWHAZ housekeeping gene was used as a normalization control in all qPCR reactions, as its expression was not affected upon viral infection.

### 2.12. ELISA Assay

Cell supernatants were collected and supplemented with 30 μM EDTA and 110 μM L-ascorbic acid to preserve dopamine in its reduced form. Cell debris was removed by centrifugation of the samples at 2000× *g* for 20 min at 4 °C. Dopamine concentration in cell supernatants was measured with the Dopamine ELISA kit (IBL International, Hamburg, Germany), as instructed by the manufacturer’s protocol.

### 2.13. Chemicals

L-3,4-dihydroxyphenylalanine (L-Dopa), 5-Hydroxytryptophan (5-HTP), dopamine (DA), serotonin (5-HT), clorgyline, phenelzine, reserpine, forskolin, phorbol 12-myristate 13-acetate (PMA), reduced L-glutathione (GSH) and L-ascorbic acid, were acquired from Sigma-Aldrich (St. Louis, MO, USA). Nepicastat, norepinephrine (NE) and prochlorperazine (PCZ) were obtained from Cayman Chemical (Ann Arbor, MI, USA).

### 2.14. Statistical Analysis

In each diagram, mean values calculated from no less than three independent experiments in triplicate are represented by bars, and standard deviations are indicated by error bars. Experimental data was analyzed using unpaired Student’s *t*-test and statistical significance was considered by *p* values less than 0.05. Statistical calculations were made with Excel Microsoft Office^®^ (Microsoft Corporation, Redmond, WA, USA) or Prism (Graphpad Software, Inc., San Diego, CA, USA).

## 3. Results

### 3.1. DDC Silencing Positively Affects DV Replication and Attenuates the Infection Related Cell Death

Our previous work [21] using the DDC inhibitor, carbidopa, has suggested that the enzymatic activity of DDC plays important role in the downregulation of DV replication; however, carbidopa also positively affected both the intracellular ATP levels and AKT phosphorylation. As DV infection produces a cytopathic effect, at least part of the virus upregulation upon treatment with carbidopa was related to the AKT activation-mediated cells survival enhancement. In the same study, the use of non-cytotoxic concentrations of DDC substrates was found to reduce viral replication [21]; however, direct evidence of the DDC-mediated conversion of substrates to products was missing. To circumvent these problems, in the present study, we performed *DDC* gene silencing and addressed the effect of DDC substrates on DV replication.

**Figure 1:**
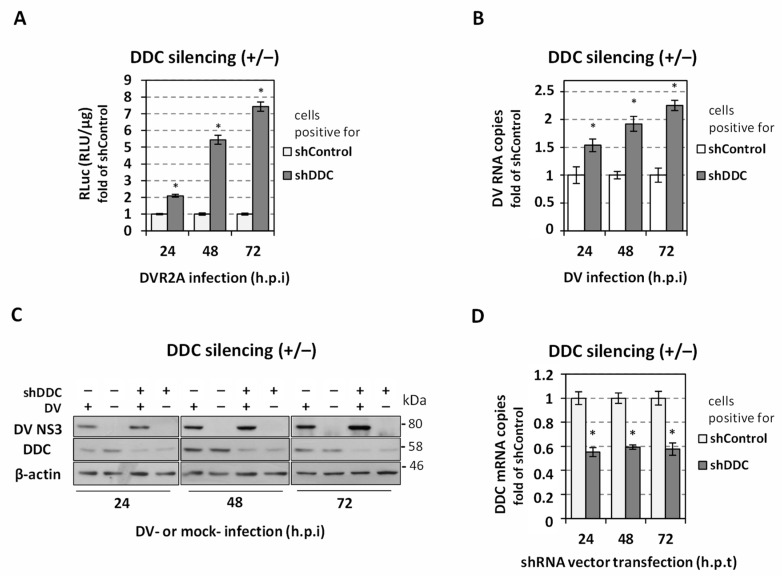
*DDC* silencing enhances DV genome replication. Twenty-four hours after electroporation of Huh7 cells with the shDDC vector, that expresses an shRNA targeting DDC mRNA, or the shControl vector, cells were infected by DV (DV-2 16681 strain, at an MOI = 0.1) or DVR2A reporter virus (MOI = 0.1) for 4 h, and after virus inoculum removal was further cultured for the indicated hours post-infection (h.p.i). (**A**) Levels of Renilla luciferase activity (RLuc)*,* indicative of DV replication, were expressed as RLU/μg of total protein amount. (**B**) RT-qPCR analysis was performed to determine DV plus-strand RNA levels. At each time point, values derived from shControl-expressing cells were set to one. Data shown are means ± standard deviations of values from three independent experiments in triplicate. * *p* < 0.001 vs. shControl. (**C**) SDS-PAGE and immunoblot analysis were performed using lysates from cells that were first transfected (via electroporation) with shDDC (+) or shControl (−) vectors and then infected with DV (+) or mock-infected (−). Antibodies detecting DV NS3, DDC and β-actin proteins were used. β-actin served the purpose of loading control. An experiment that is representative of three biologically independent repetitions is shown. (**D**) DDC mRNA amounts were quantified by RT-qPCR in cells transfected with the shDDC vector or the shControl vector, DDC mRNA levels were normalized to the mRNA levels of the housekeeping gene (YWHAZ). At each time point, values derived from cells expressing shControl were set as one. The mean values ± standard deviations from three biologically independent transfection experiments in three technical replicates are presented. * *p* < 0.001 vs. shControl.

As a first step, we examined how the suppression of *DDC* expression affects the replication of DV genome. For this purpose, hepatic Huh7 cells were transfected via electroporation with the lentiviral plasmid vector expressing an shRNA, which targets DDC mRNA (shDDC), or with the corresponding control vector (shControl). Consequently, 24 h’ post-transfection (h.p.e), cells were inoculated with DV-2 (strain 16681) or the reporter DV virus (DVR2A) that expresses Renilla luciferase, at a multiplicity of infection (MOI) of 0.1, for 4 h. After medium replacement, cells were incubated for 24, 48, or 72 h, then the cells were lysed and the levels of DV replication-derived Renilla luciferase (RLuc) activity or DV RNA levels were determined. The silencing of *DDC* significantly enhanced the replication of DV as shown by RLuc activity and the levels of DV RNA positive strand (Figure 1A,B). Consistently, a significant accumulation of DV NS3 protein was detected upon *DDC* silencing (Figure 1C). The downregulation of DDC protein and mRNA in shDDC- as compared to shControl-expressing cells was confirmed (Figure 1C,D). Replication kinetics of the DV and DVR2A virus in shControl-expressing cells are shown in Figure S1A,B.

**Figure 2:**
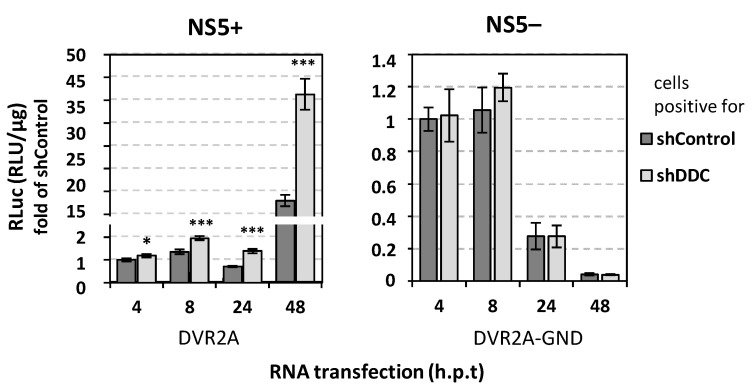
*DDC* silencing affects DV genome replication but not translation. Huh7.5 cells stably expressing shDDC or shControl were transfected with the in vitro transcribed RNA derived from the subgenomic reporter pFK-sgDVR2A (NS5+) (left panel), or its replication deficient mutant pFK-sgDVR2A-GND (NS5−) (right panel) and then cultured for the indicated time points. Then, the cells were lysed and Renilla luciferase enzymatic activity was quantified. Values were normalized to the ones obtained at 4 h and expressed as fold of the ones obtained in shControl-expressing cells. Data shown are means ± standard deviations of values from three biologically independent transfection experiments in three technical replicates. Values derived from shControl-expressing cells were set as one. * *p* < 0.05, *** *p* < 0.001 vs. shControl.

To determine the exact stage of DV replication cycle that is influenced by *DDC* silencing, Huh7.5 cells stably expressing the shDDC that suppresses *DDC* expression (Figure S2A) or the shControl, were transfected with the in vitro transcribed RNA of the subgenomic reporter sgDVR2A (NS5+) or its replication-defective variant, sgDVR2A-GND (NS5-). Cells were further cultured for the indicated time points and viral replication-derived luciferase activity was determined. *DDC* silencing was found to enhance sgDVR2A replication, while no difference was observed in the case of sgDVR2A-GND, excluding the possibility that the effect of DDC on DV is exerted at the step of virus translation (Figure 2). These data clarified that *DDC* silencing affects specifically the genome replication of the virus, without affecting viral translation.

**Figure 3:**
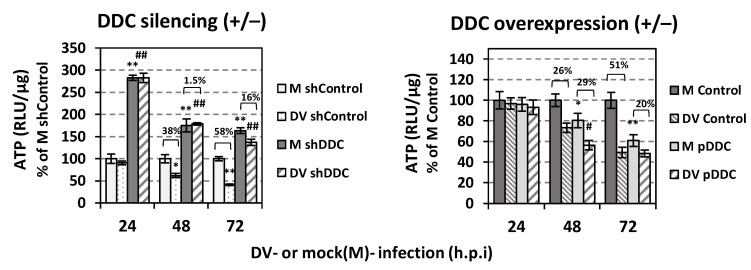
The expression levels of DDC affect the intracellular ATP of DV-infected cells. (**Left panel**) *DDC* silencing increases the intracellular ATP levels of DV-infected cells. Huh7 cells were transfected with shDDC vector or the control vector (shControl), (via electroporation) 24 h.p.t. were inoculated with non-reporter DV (MOI = 1) virus or were mock-infected (mock, M) for 4 h and then cultured for the pointed hours post-infection (h.p.i). shControl-expressing mock-infected cell (M shControl) values were set as 100%. The mean values ± standard deviations from three independent experiments in triplicate are featured. Percentages of AΤP reduction are shown above brackets. * *p* < 0.01, ** *p* < 0.001 vs. Μ shControl, ## *p* < 0.001 vs. DV shControl. (**Right panel**) DDC overexpression reduces the intracellular ATP levels of DV-infected cells. Twenty-four hours after transfection of Huh7 cells with pcDNA3.1-DDC (pDDC) (+) or the control vector (Control) (−), cells were infected with non-reporter DV (MOI = 1) virus or mock-infected (mock, M) for 4 h and cells were lysed at the indicated h p.i. By the use of a chemiluminescence-based assay the intracellular ATP was quantified and calculated as RLU/μg of total protein amount. Values from cells transfected with the control vector and mock-infected (M Control) were set as 100%. * *p* < 0.01, ** *p* < 0.001 vs. Μ Control, # *p* < 0.01 vs. DV Control.

Next, we aimed to determine the effect of *DDC* silencing on DV-mediated reduction of cell viability. For this, we quantified intracellular ATP levels. As shown in Figure 3-left panel, DDC silencing caused an increase in the ATP amount of mock-infected cells and abrogated DV-mediated reduction in cell viability for up to 72 h p.i., suggesting an implication of DDC in cell death, in agreement with our previous work concerning the negative effect of DDC on PI3K/AKT activity [25]. To further characterize the association of DDC expression with the infected cell viability, we performed transfection of Huh7 cells, via electroporation, with a mammalian plasmid vector that overexpresses DDC [53,54], or with a control vector. At 24 h.p.t. we infected the cells with DV (DV-2 16681 strain) or not (mock-infected), and further incubated them for 24–72 h. In agreement with the above results, in cells overexpressing DDC, the intracellular ATP levels at 48–72 h.p.i. were greatly decreased in both infected and mock-infected cells, compared to cells transfected with the control vector, and DV-induced cell death was exacerbated as early at 48 h.p.i. (Figure 3-right panel).

### 3.2. DDC Silencing Reduces the Antiviral Activity of DDC Substrates

**Figure 4:**
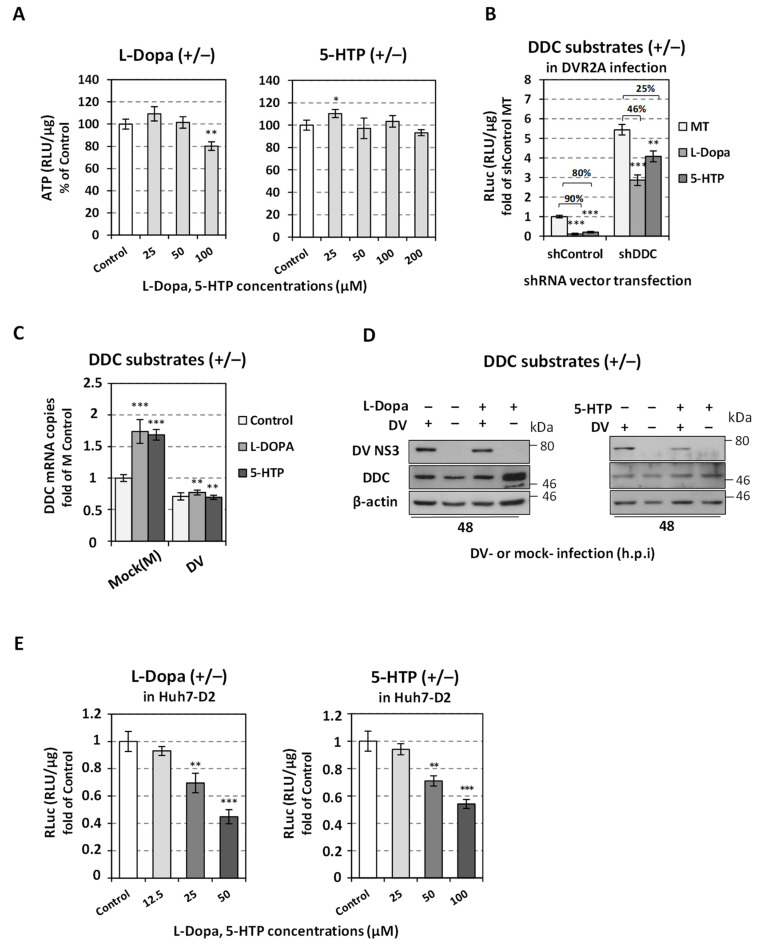
The application of DDC substrates negatively affects the replication of DV. (**A**) Evaluation of different concentrations of DDC substrates concerning their cytotoxicity of DV-infected cells. Huh7 cells were transfected with the shControl vector (via electroporation) and 24 h.p.t. were inoculated with DV (MOI = 0.1) for 4 h. After virus inoculum removal, cells were treated with different concentrations of L-Dopa (left panel) or 5-HTP (right panel), or were mock-treated (Control), for 48 h. Then the cells were lysed and by the use of a chemiluminescence-based assay, the intracellular ATP was quantified. Infected mock-treated cell (Control) values were expressed as one. * *p* < 0.05, ** *p* < 0.01 vs. Control (**B**) *DDC* silencing reduces the effect that the DDC substrates have on DV replication. Huh7 cells were transfected with the shDDC vector or the control vector (shControl). Twenty-four h.p.t., cells were infected with DVR2A (MOI = 0.1) for 4 h and after virus inoculum removal were treated with L-Dopa (50 μΜ) or 5-HTP (100 μΜ), or were mock-treated (MT), and further cultured for 48 h. Dengue replication-derived Renilla luciferase activity was expressed as RLU/μg of total protein amount. Values are compared to the ones of MT shControl-expressing DVR2A-infected cells. Percentage values over the brackets represent the fold difference of luciferase activity levels between the DDC substrate-treated cells and MT cells. ** *p* < 0.01, *** *p* < 0.001 vs. MT. (**C**,**D**) DDC substrates enhance the expression of DDC in mock-infected cells. Huh7 cells were transfected with the shControl vector via electroporation, 24 h.p.t. were inoculated with DV-2 16681 (MOI = 1) or were mock-infected (M) for 4 h and then treated with L-Dopa (50 μΜ) or 5-HTP (100 μΜ), or were mock-treated (Control) for 48 h. (**C**) DDC mRNA levels were quantified (RT-qPCR) and mock-infected mock-treated ((mock (M) Control)) cell values were set as one. The mean values ± standard deviations from three independent experiments in triplicate are presented. ** *p* < 0.01, *** *p* < 0.001 vs. M Control. (**D**) Lysates of DV (+) or mock (−) infected cells that had been treated (+) with L-Dopa (50 μΜ) or 5-HTP (100 μΜ) or mock-treated (−) for 48 h, were subjected to SDS-PAGE and immunoblot analysis, with the use of antibodies against DV NS3, DDC and β-actin (loading control) proteins. (**E**) DDC substrates reduce the replication of DV replicon. The Huh7-D2 stable cell line that harbors the DV subgenomic replicon was treated with the indicated concentration of L-Dopa (left panel) or 5-HTP (right panel) or was mock-treated (Control) for 48 h. Subgenomic replicon-derived Renilla luciferase activity was determined by chemiluminescence-based assay. Control cell values were set as one. Data shown are means ± standard deviations of values from three independent experiments in triplicate. ** *p* < 0.01, *** *p* < 0.001 vs. Control.

In order to examine the antiviral effect of the conversion of DDC substrates to products on DV, Huh7 cells were transfected, via electroporation, with the shDDC or the control vector (shControl), 24 h later were inoculated with the reporter DVR2A (or mock-infected) for 4 h, and subsequently were treated with non-cytotoxic concentrations of L-Dopa or 5-HTP, based on the intracellular ATP amounts (Figure 4A). In parallel, cells treated with the respective solvent (mock-treated cells, MT) were used as a control. As shown in Figure 4B, *DDC* silencing significantly attenuated the negative effect of both L-Dopa and 5-HTP on DV replication, as detected by reporter virus-derived luciferase levels. This suggests that DDC enzymatic activity is a prerequisite for the effect of DDC substrates on DV replication and verifying that L-Dopa and 5-HTP are transported inside hepatocytes. The effect of L-Dopa and 5-HTP on viral replication was confirmed at the protein level, as shown by the reduction caused in the viral NS3 protein (Figure 4C). The treatment of Huh7 cells with L-Dopa and 5-HTP enhanced the expression of DDC mRNA and protein (Figure 4C,D) as compared to mock-infected mock-treated cells (M Control), possibly due to feedback mechanisms; however; this enhancement was not observed in DV-infected cells. This may possibly suggest that DV somehow inhibits the mechanism of regulation that coordinates the expression of DDC with the quantity of the substrates of this enzyme. The negative impact on viral replication was also obtained when the above DDC substrates were applied to Huh7-D2 cells (Figure 4E), harboring DV-2 16681 subgenomic replicon, verifying the important role of DDC enzymatic activity on DV replication.

### 3.3. External Application of the DDC Products Reduces DV Replication

**Figure 5:**
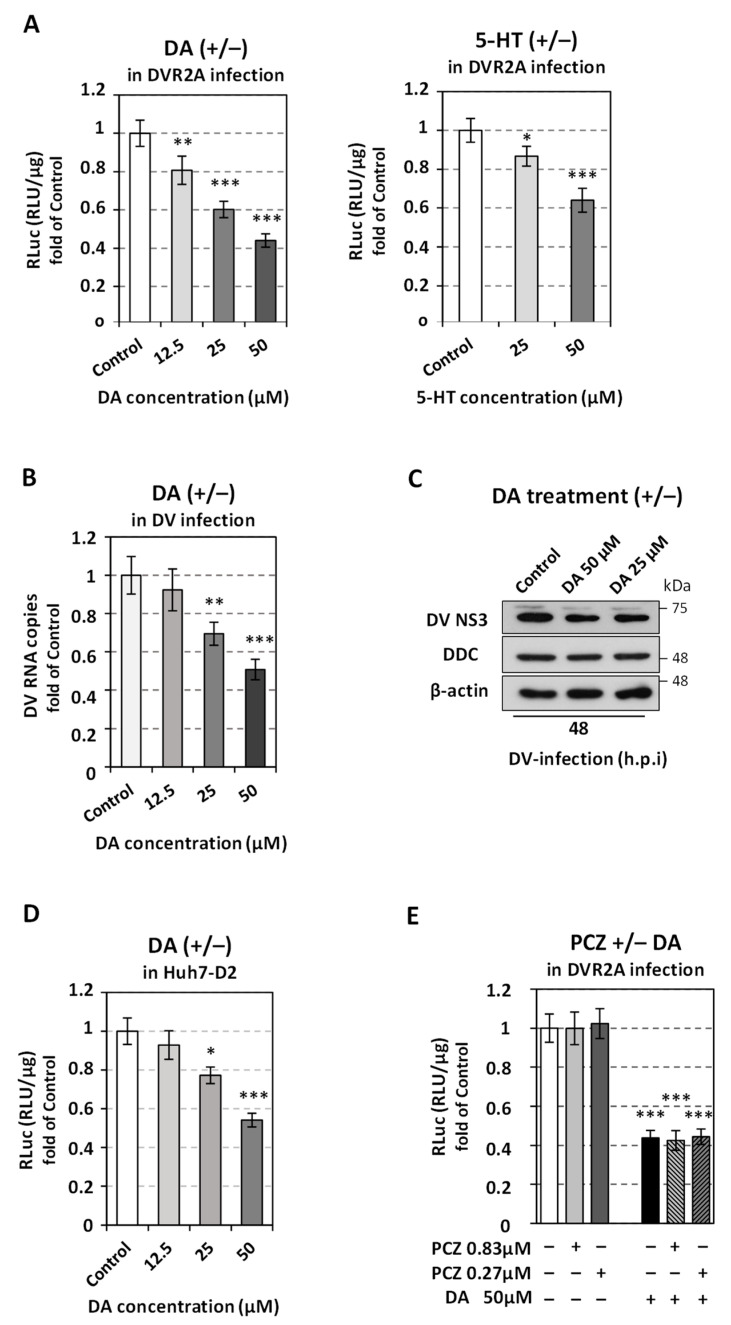
DDC enzyme activity products reduce DV replication. Huh7 cells were inoculated with DVR2A (MOI = 0.1) (**A**,**E**) or DV (MOI = 0.1) (**B**,**C**) for 4 h, and subsequently treated with the pointed concentrations of DA, 5-HT and/or PCZ, for 48 h. Mock-treated cells were used in parallel. (**A**) RLuc activity, indicative of viral replication, was quantified in cells cultured in the presence of DA (left) or 5-HT (right), or in mock-treated cells (Control). (**B**) RT-qPCR analysis was performed to determine DV plus-strand RNA levels in cells treated with DA or mock-treated (Control). (**A**,**B**) Control cell values were set as one. Data shown are means ± standard deviations of values from three independent experiments in triplicate. * *p* < 0.05, ** *p* < 0.01, *** *p* < 0.01 vs. Control. (**C**) SDS-PAGE and immunoblot analysis were carried out in lysates of DV-infected cells treated for 48 h with 25 μM or 50 μM DA, or mock-treated (Control), with the use of antibodies against DV NS3, DDC, or β-actin (loading control) proteins. An experiment that is representative of three independent repetitions is shown. (**D**) The Huh7-D2 cell line was treated for 48 h with the pointed concentrations of DA or mock-treated (Control). Levels of Renilla luciferase activity were calculated as RLU/μg of total protein amount. (**Ε**) Dopamine affects DV replication possibly through its uptake and not via its receptor signaling. Effect of DA and PCZ combination on DVR2A replication. DV-infected mock-treated cell (Control, (−)) values were expressed as one. (**D**,**E**) The mean values ± standard deviations from three biologically independent experiments in triplicate are presented. * *p* < 0.05, *** *p* < 0.001 vs. Control (−).

The above results implied that DDC enzyme activity-derived dopamine and serotonin exerted a negative effect on DV replication. Thus, we analyzed further the effect of catecholamines and serotonin, as well as the role of enzymes/proteins implicated in catecholamines biosynthesis, storage and degradation, on DV replication. First, we examined whether the treatment of cells with the DDC products dopamine (DA) and serotonin (5-HT) affects DV. Huh7 cells were inoculated with DVR2A for 4 h, and, after virus inoculum withdrawn, were treated with non-cytotoxic concentrations of DA or 5-HT, or were mock-treated (Control), for 48 h. As shown in Figure 5A (see also Figure S3A,B for cytotoxic effects of DA and 5-HT), both DA and 5-HT lessened the virus-derived RLuc activity by ~2-fold. Accordingly, a decrease was observed on viral RNA (Figure 5B) and protein (Figure 5C) levels, after treatment with DA, compared to Control cells. The negative impact of DA on viral RNA replication was confirmed in Huh7-D2 cell line which harbors DV subgenomic replicon (Figure 5D). DDC protein and mRNA levels, in both infected and mock-infected cells, were not notably altered by the external application of DA (Figure S4A). DA slightly increased (1.35-fold) the mRNA levels of the downstream biosynthetic enzyme dopamine β-hydroxylase (DBH), which uses DA as a substrate (Figure S5A). This could suggest a positive regulation of *DBH* gene expression, or DBH mRNA stability, by the substrate of the enzyme. To elicit if the mechanism, through which DA negatively influences DV, involves dopaminergic receptor signaling, we applied, to DVR2A-infected cells, DA in combination with prochrorperazine (PCZ), which is an antagonist of the well characterized D2 dopamine receptor that is known to be expressed in hepatocytes [62,63,64], and analyzed the effect on viral replication. As shown in Figure 5E (see also Figure S3C for cytotoxic effects of PCZ) PCZ had no effect on DV either we applied DA to cells or not. Combining the aforementioned data indicate that the effect of the exogenously supplied dopamine on DV is possibly due to the uptake of DA and not via its receptor signaling.

### 3.4. Augmentation of DV Replication by the Inhibition of the Monoamine Transporter (VMAT) and the Blockage of Dopamine Uptake

The role of catecholamine uptake and storage on DV replication was directly studied by suppressing VMAT2 activity with the irreversible inhibitor reserpine [65]. VMAT2 is the main monoamine transporter in the liver [19,20,66]. VMAT2 regulates the catecholamines and serotonin uptake from extracellular sources through upregulation of their transporters, and is also responsible for their entry in storage vesicles, where DBH converts DA to NE [26,27,28,29]. Reserpine is known to deplete intracellular catecholamine stores [67,68] by suppressing the expression [69,70] and activity [29] of dopamine and norepinephrine transporters and consequently the uptake of catecholamines. This occurs through a mechanism dependent on VMAT- and VMAT-containing catecholamine storage vesicles [26,27,28,29].

**Figure 6:**
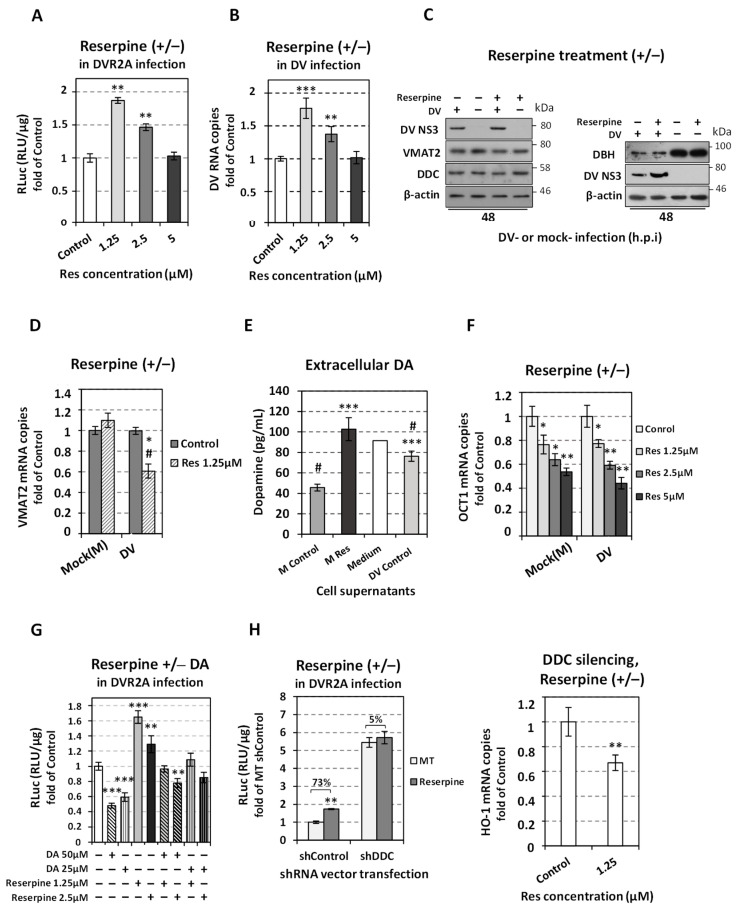
Effect of VMAT-inhibitor reserpine on DV replication. (**A**–**G**) Huh7 cells were inoculated with the reporter DVR2A (MOI = 0.1) ((**A**,**G**,**H**) left panel) or DV (MOI = 0.1) (**B**–**F**) virus, or were mock-infected (mock, M), for 4 h and then treated with reserpine (Res), or mock-treated (Control) for 48 h. (**A**,**B**) For different concentrations of reserpine applied: (**A**) Levels of Renilla luciferase activity, indicative of DV replication, were calculated as RLU/μg of total protein amount. ** *p* < 0.001 vs. Control. (**B**) RT-qPCR analysis was performed to determine DV plus-strand RNA levels. Control mock-infected cell values were set as one. The mean values ± standard deviations from three independent experiments in triplicate are presented. ** *p* < 0.01 vs. Control, *** *p* < 0.001 vs. Control. (**C**,**D**) The result of reserpine treatment on DV NS3 and VMAT2 protein expression. Huh7 cells were infected with DV (MOI = 0.1) or not (mock-infected) for 4 h and then were treated with reserpine (1.25 μΜ) or mock-treated for 48 h. (**C**) SDS-PAGE and immunoblot analysis were carried out in lysates of DV-infected (+) or mock-infected (−) cells treated with reserpine (1.25 μΜ) or mock-treated (−) for 48 h using antibodies to detect DV NS3, VMAT2, DDC, and β-actin (loading control) proteins. An experiment that is representative of three independent repetitions is shown. (**D**) RT-qPCR was used for the quantification of VMAT2 mRNA levels. Mock-infected mock-treated (M Control) cell values were set as one. * *p* < 0.001 vs. M Control, # *p* < 0.001 vs. DV Control. (**E**) Effect of reserpine on DA uptake by cells. 1.25 μΜ reserpine was used to treat Huh7 cells for 48 h, either in the absence (mock-infected, M) or presence of DV infection (MOI = 0.1), and then cell supernatants were collected. Mock-treated (Control) were used in parallel. Dopamine levels in collected supernatants were measured with a non-competitive enzyme immunoassay and compared to the ones in plain culture medium (Medium). *** *p* < 0.001 vs. M Control, # *p* < 0.001 vs. Medium. (**F**) Effect of reserpine on *OCT1* expression. After a 4 h inoculation of Huh7 cells with DV (MOI = 0.1), cells were treated with different concentrations of reserpine or were mock-treated (Control) for 48 h, and then cells were lysed. OCT1 mRNA amounts were quantified by RT-qPCR and normalized to the mRNA levels of the housekeeping gene. Control cell values were set as one. * *p* <0.01, ** *p* <0.001 vs. Control. (**G**) Effect of combinatory treatment with reserpine and DA on DVR2A replication. Renilla luciferase activity, was quantified in cell lysates. Mock-treated cell (Control, (−)) values were set as one. ** *p* <0.01, *** *p* <0.001 vs. Control (−). (**H**) *DDC* silencing attenuates the effect of reserpine on DV replication. After a 4 h inoculation of Huh7.5 cells stably expressing shDDC or shControl with DVR2A (MOI = 0.1) virus, cells were treated with reserpine (1.25 μΜ) or were mock-treated (MT), and further cultured for 48 h. ((**H**) left panel) Levels of Renilla luciferase activity were expressed as RLU/μg of total protein amount. Values are compared to the ones of shControl-expressing mock-treated (shControl MT) cells. Data shown are means ± standard deviations of values from three biologically independent experiments in triplicate. Percentages of viral replication induction by treatment, are shown above brackets. ((**H**) right panel) Reserpine treatment reduces the expression of oxidative stress-related genes in cells expressing shDDC. RT-qPCR quantification of HO-1 mRNA levels in Huh7.5 cells stably expressing shDDC that were treated with 1.25μM reserpine or were mock-treated (Control). Control cell values were set as one. The mean values ± standard deviations from three independent experiments in triplicate are featured. ** *p* < 0.01 vs. Control.

To date, few lines of evidence have shown that VMAT2 is present in normal human liver tissue [19,20]. Here, we identified by RT-qPCR the presence of VMAT2 mRNA in Huh7 cells, whereas, VMAT1 mRNA levels were not detectable (data not shown). This is consistent with the tissue specificity of VMAT1 expression [66] and with our previous results in Huh7.5 cells [33]. In addition, western blot analysis confirmed the expression of VMAT2 protein (Figure 6). Therefore, we sought to identify the effect of VMAT2 inhibition on DV replication, using non-cytotoxic concentrations of reserpine (Res) according to the intracellular AΤΡ levels (Figure S3D); for this, Huh7 cells were infected with DV or the reporter DVR2A virus and further cultured in the presence of different concentrations of reserpine for 48 h. A positive effect of VMAT2 inhibition on DV replication was detected, as shown by the upregulated levels of DVR2A-derived luciferase activity (Figure 6A), DV RNA (Figure 6B) as well as DV NS3 protein (Figure 6C). Furthermore, a similar rise of DV replication was also observed in Huh7-D2 cells harboring the DV subgenomic replicon (Figure S6A) upon reserpine use. Interestingly, the most significant positive effect on DV replication was apparent in the lowest concentration (1.25 μΜ) of reserpine applied.

Reserpine did not affect the levels of VMAT2 in mock-infected cells; however, in DV-infected cells, the levels of VMAT2 protein (Figure 6C left) and mRNA (Figure 6D) were decreased upon reserpine treatment. Additionally, in reserpine-treated cells, an increase was exhibited in extracellular dopamine levels, which stood approximately at 2.25-fold more than in Control (mock-treated) cells and was comparable to the ones in plain culture medium not exposed to cells (Medium), as demonstrated by ELISA assay using cell supernatants (Figure 6E). This data is consistent with previous studies [71] indicating that cells uptake/metabolize the medium-containing dopamine, whereas reserpine appeared to completely nullify this process. To further elucidate the negative impact of reserpine on dopamine uptake, we measured the transcription of *OCT1*. This gene encodes the major catecholamine transporter in hepatocytes [72,73], which also transports a variety of substrates, including L-Dopa [74]. Indeed, reserpine reduced OCT1 mRNA levels in both infected and mock-infected cells as determined by qPCR (Figure 6F), while OCT1 mRNA levels were independent of the presence of the virus (Figure S7A). This agrees with respective data obtained in the case of HCV-infected and mock-infected Huh7.5 cells [33] and suggests that the reserpine-mediated downregulation of *OCT1* may produce an additive effect on the decrease of the intracellular levels of catecholamines. In agreement, reserpine diminished the negative impact of externally applied dopamine on DV replication (Figure 6G); moreover, upon *DDC* silencing, reserpine failed to affect DV replication (Figure 6H left) while causing a decrease in antioxidant gene HO-1 mRNA levels (Figure 6H right), indicating a more reducing environment.

Reserpine treatment did not affect the expression of DDC in mock-infected cells. In DV-infected cells reserpine reduced DDC mRNA levels (Figure S4B), possibly due to the reserpine-mediated enhancement of virus replication that subsequently downregulates DDC; moreover, reserpine did not affect the levels of DBH protein, in both DV-infected and mock-infected cells (Figure 6C right), nor altered the DBH mRNA levels in mock-infected cells (Figure S5B). As in the case of HCV [33], DV infection substantially reduced the expression of this enzyme (Figure 6C right). This implies that the amount of DBH is somehow associated with the amount of DDC. In agreement, it has been shown that the expression of TH, DDC and DBH can be coordinated in the cell [75].

### 3.5. The Implication of the Norepinephrine Biosynthetic Enzyme DBH in DV Replication

**Figure 7:**
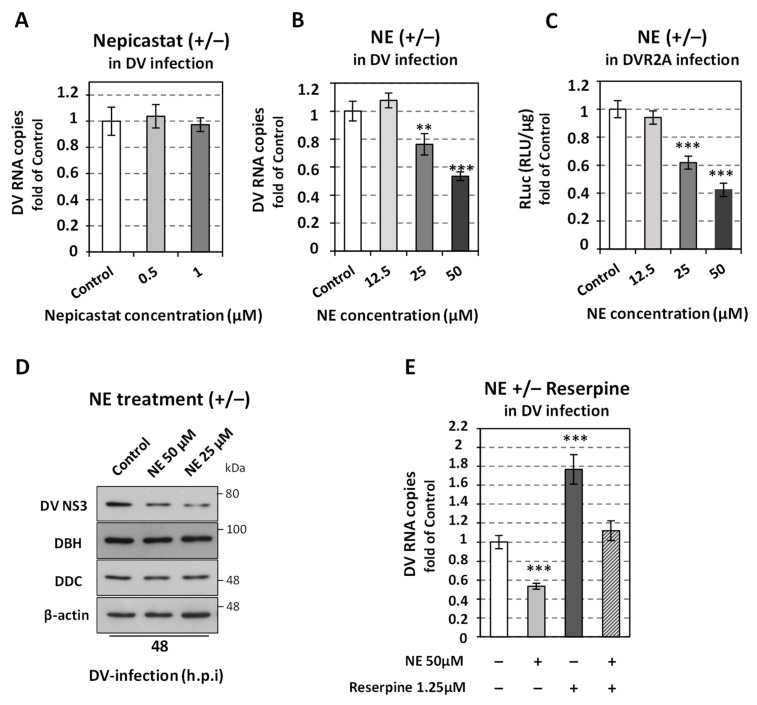
DBH inhibition (nepicastat) has no effect on DV replication, in contrast, to the inhibitory effect exerted by the DBH product, NE. After a 4 h inoculation of Huh7 cells with the non-reporter DV (MOI = 0.1) (**A**,**B**,**D**,**E**) or reporter DVR2A (MOI = 0.1) (**C**) and subsequent inoculum removal, cells were treated with different concentrations of nepicastat (**A**) or NE (**B**–**E**) or were mock-treated (Control) for 48 h. (**A**,**B**) RT-qPCR analysis was performed to determine DV plus-strand RNA levels in cells subjected to nepicastat (**A**) or NE (**B**) treatment, or in mock-treated (Control) cells. ** *p* < 0.01, *** *p* < 0.001 vs. Control. (**C**) Renilla luciferase activity, indicative of dengue virus replication, was determined in NE treated or mock-treated (Control) cells. Infected mock-treated cell values were set as one. The mean values ± standard deviations from three independent experiments in triplicate are featured. *** *p* < 0.001 vs. Control. (**D**) SDS-PAGE and immunoblot analysis were carried out in lysates of cells infected by DV and subsequently treated with NE or mock-treated (Control) for 48h. Antibodies detecting DV NS3, DBH, DDC or β-actin (loading control) proteins were used. An experiment that is representative of three independent repetitions is presented. (**E**) Effect of co-treatment with NE and reserpine on DV genome replication. RT-qPCR analysis was utilized to quantify plus-strand RNA levels of DV that were then normalized to the mRNA levels of the housekeeping gene. Infected mock-treated cell (Control, (−)) values were set as one. Data shown are means ± standard deviations of values from three independent experiments in triplicate. *** *p* < 0.001 vs. Control (−).

Next, we further analyzed the effect of catecholamine pathway on DV virus replication by focusing on the downstream of DDC biosynthetic enzyme dopamine β-hydroxylase (DBH), that synthesizes NE. To analyze the effect of DBH activity on the virus, Huh7 cells were infected with DVR2A, and subsequently treated with nepicastat, a selective DBH inhibitor [76]. Non-cytotoxic concentrations of nepicastat were used according to the intracellular AΤΡ levels (Figure S3E). Interestingly, as shown in Figure 7A, nepicastat did not appear to influence DV RNA replication or the replication-indicative RLuc activity and protein levels (Figure S8A,B). Additionally, nepicastat did not influence the DDC mRNA levels either (Figure S4C). On the other hand, treatment of cells with NE, reduced viral replication based on the viral RNA, luciferase assay and protein levels (Figure 7B–D); however, it enhanced the intracellular ATP content in DV-infected and mock-infected cells (Figure S3F), possibly due to upregulation of aerobic glycolysis [77,78]. The negative impact of NE on viral replication was also confirmed in the cell line that harbors the DV subgenomic replicon (Figure S6B). Furthermore, external administration of reserpine, which has been shown to act as an inhibitor of NE uptake [29,69], attenuated the adverse effect of NE on DV replication (Figure 7E). This is consistent with our finding for the combinatory treatment of dopamine and reserpine (Figure 6G). Finally, DBH levels were not affected by NE (Figure 7D), as DA did not affect the expression of *DDC* (Figure 4C,D), while NE reduced DDC mRNA levels (Figure S4D). The above data suggest that, as in the case of HCV [33], the NE uptake and accumulation in the cell cytosol constitute a negative factor for DV replication, whereas its restriction inside the cytosolic vesicles diminishes its effect.

### 3.6. Effect of Inhibition of MAO Monoamine Degradation Enzymes on DV Replication

**Figure 8:**
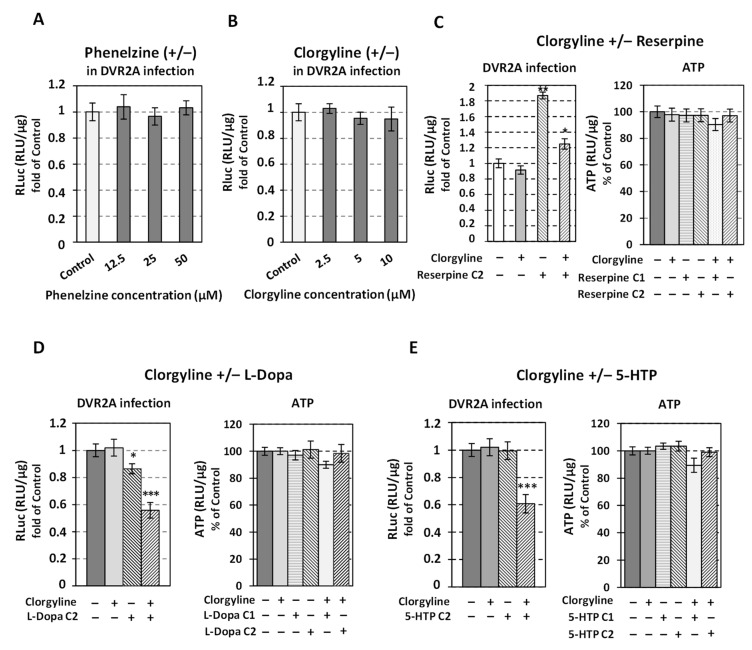
Elevated catecholamine levels make DV replication sensitive in MAO inhibitors. Effect of non-cytotoxic concentrations of MAO inhibitors on DV replication. ((**A**–**E**) left panels): After a 4 h inoculation of Huh7 cells with DVR2A (MOI = 0.1), cells were treated with different concentrations of MAO inhibitors (**A**,**B**), or co-treated with clorgyline and reserpine (**C**), clorgyline and L-Dopa (**D**), clorgyline and 5-HTP (**E**), or were mock-treated (Control, (−)), for 48 h. Cells were then lysed and levels of Renilla luciferase activity were measured. Control cell values were set as one. ((**C**–**E**) right panels): Intracellular ATP levels were determined. Mock-treated cell (Control, (−)) values were set as 100%. For Clorgyline = 5 μΜ, Reserpine: C1 = 2.5 μΜ, C2 = 1.25 μΜ, L-Dopa: C1 = 30 μΜ, C2 = 15 μΜ and for 5-HTP: C1 = 50 μΜ, C2 = 25 μΜ. In all panels, the mean values ± standard deviations from three independent experiments in triplicate are presented. * *p* < 0.05, ** *p* < 0.01, *** *p* < 0.001 vs. Control (−).

**Figure 9:**
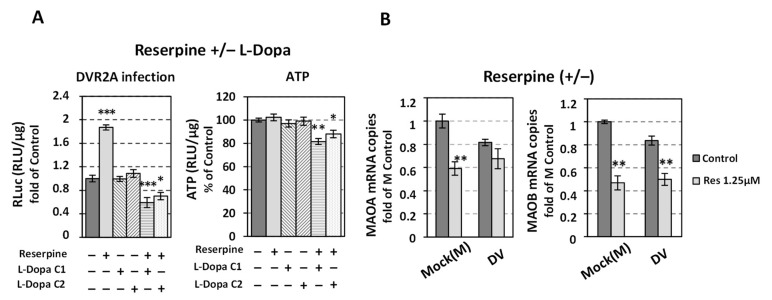
Reserpine increases the sensitivity of DV replication in DDC substrates. (A): After a 4 h inoculation of Huh7 cells with DVR2A (MOI = 0.1), cells were treated with reserpine 1.25 μΜ in the presence or not of L-Dopa or were mock-treated (Control, (−)), for 48 h. ((**A**)-Left): Cells were then lysed and Renilla luciferase activity was determined. Infected control cell values were set as one. ((**A**)-Right): Intracellular ATP levels were determined. Control cell values were set as 100%. For L-Dopa: C1 = 15 μΜ and C2 = 7.5 Μμ. Error bars indicate standard deviations. * *p* < 0.05, ** *p* < 0.01, *** *p* < 0.001 vs. Control. (**B**) Reserpine negatively affects the expression of *MAO* genes. Huh7 cells were inoculated with DV (MOI = 1) or were mock-infected (mock, M) for 4 h, and subsequently treated with reserpine (1.25 μΜ) or mock-treated (Control) for 48 h. RT-qPCR analysis was performed to determine the mRNA amounts of MAO-A and MAO-B and the mRNA of the housekeeping gene (YWHAZ) was used for normalization. M Control cell values were expressed as one. The mean values ± standard deviations from three independent experiments in triplicate are presented. ** *p* < 0.001 vs. M Control.

Next, based on the negative effect of the catecholamine biosynthesis on DV, we aimed to characterize the relationship of the downstream metabolic pathway with the virus. Monoamine oxidases A and B (MAO-A and B) are the enzymes involved in the oxidative degradation process of monoamines, including dopamine, norepinephrine, and serotonin. To investigate a putative effect of MAO enzymatic activity on DV proliferation, we used the non-selective and irreversible MAO inhibitor phenelzine [79], as well as the selective and irreversible MAO-A inhibitor clorgyline (Figure S3G, cytotoxicity profile) [80]. DVR2A-infected cells were treated for 48 h with different, non-cytotoxic concentrations of the inhibitors (or mock-treated) and then the Renilla luciferase activity, which acts as a viral replication indicator, was determined. As shown in Figure 8A,B, neither phenelzine nor clorgyline altered DV replication, something also confirmed in the case of DV subgenomic replicon cell line Huh7-D2 (Figure S6C). The fact that MAOs are downstream of DDC in the metabolic pathway of biogenic amines prompted us to examine whether clorgyline has a putative feedback inhibitory effect on the pathway. For this, in clorgyline-treated cells we determined the levels of DDC mRNA and protein. As we observed, clorgyline did not alter them (Figure S4E,F), in accordance with previous results [81]; however, when DV-infected cells were co-treated with clorgyline and reserpine, that inhibits the entry of catecholamines in the cell and the cell cytoplasmic vesicles, a downregulation of DV replication was observed compared to the ones acquired with reserpine alone (Figure 8C). Additionally, when DV-infected cells were treated with clorgyline, in the presence of the DDC substrates L-Dopa or 5-HTP, a strong downregulation of DV replication was observed, although the cell viability was not affected (Figure 8D,E). Interestingly, in the presence of reserpine, the treatment of cells with specific concentrations of L-Dopa or 5-HTP—that when used alone did not influence DV replication nor cell viability—lowered the cell viability and strongly inhibited DV replication, as compared to Control (mock-treated) cells (Figure 9A and Figure S7B). The above results suggest that these co-treatments accumulate dopamine in the cytoplasm, inhibiting DV replication; moreover, cell cytotoxicity may arise in the case of co-treatment with reserpine that traps dopamine in the cell cytoplasm. Finally, we evaluated the effect of reserpine on *MAO* expression in mock-infected and DV-infected cells. As shown, MAO-A and -B mRNAs were reduced upon reserpine treatment in a virus infection-independent manner (Figure 9B), which may be due to the lower cytoplasmic levels of DA. Thus, MAO-A and MAO-B seem to be positively regulated by their substrates.

### 3.7. Induction of the Catecholamine and Serotonin Biosynthetic/Metabolic Pathway Downregulates DV Replication

**Figure 10:**
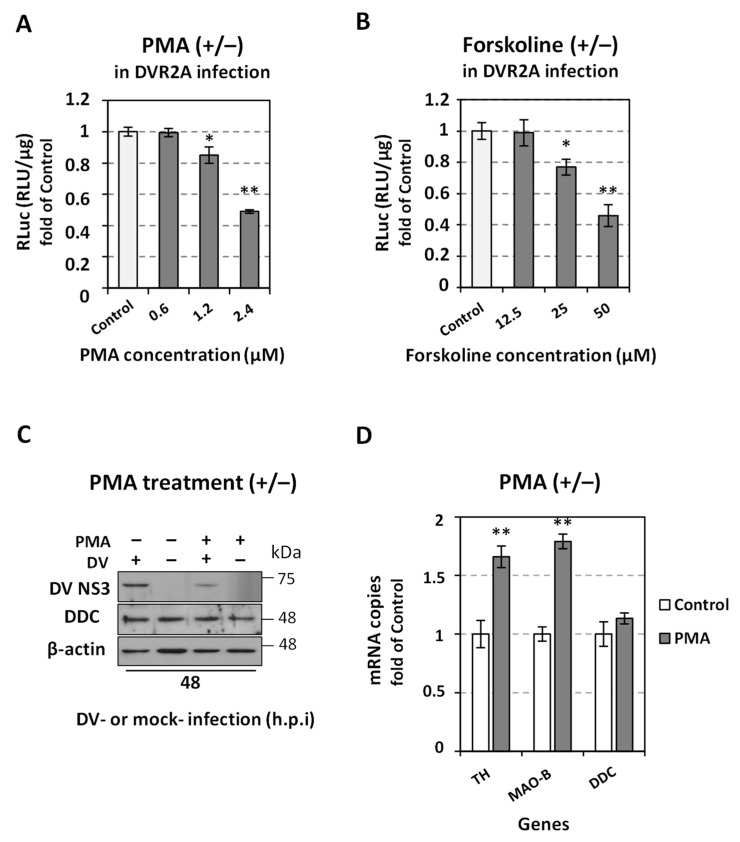
Inducers of catecholamine biosynthesis/metabolism pathway downregulate DV replication. Huh7 cells were inoculated with DVR2A (**A**,**B**) (MOI = 0.1) or DV (**C**,**D**) (MOI = 0.1), or were mock-infected (M), for 4 h and subsequently treated with the indicated concentrations of PMA (**A**,**C**,**D**) or Forskoline (**B**) or mock-treated (Control) for 48 h. (**A**,**B**) Levels of Renilla luciferase activity, indicative of dengue replication, were measured. Infected mock-treated cell values were set as one. * *p* < 0.01, ** *p* < 0.001 vs. Control. (**C**) In DV-infected (+) or mock-infected (−) cells, after virus inoculum removal, 2.4 μΜ PMA or its solvent (Control) were administrated and cells were further cultured for 48 h. SDS-PAGE and immunoblot analysis were carried out in cell lysates, with the use of antibodies against the proteins DDC and β actin (loading control). Anti-NS3 antibody was used to confirm viral infection. (**D**) Mock-infected cells were treated with PMA (2.4 μΜ) and further cultured for 48 h. Mock-treated (Control) cells were used in parallel. RT-qPCR analysis was performed to determine the mRNA amounts of TH, MAO-B and DDC and the mRNA of the housekeeping gene YWHAZ was used for normalization. Control cell values were set as one. The mean values ± standard deviations from three biologically independent experiments in triplicate are presented. ** *p* < 0.001 vs. Control.

To extend our knowledge on the bidirectional relationship that is described between the biosynthetic/metabolic pathway of biogenic amines with DV infection, two potent inducers of the pathway were examined for their putative effect on DV replication. Specifically, the phorbol 12-myristate 13-acetate (PMA) that activates the signal transduction enzyme protein kinase C (PKC), and forskolin that activates PKA (Figure S3H, cytotoxicity profile) were used. PKA and PKC activation leads to increased levels of catecholamines due to phosphorylation of DDC [82], upregulation of TH transcription and activity [12], alteration of VMAT2 vesicular trafficking [83] or increase in *MAO-B* gene expression and activity [84]. As expected, both inducers exerted a negative effect on DV replication (Figure 10A,B) that in the case of PMA was detected also by the decreased levels of DV NS3 protein (Figure 10C). An upregulation of TH and MAO-B mRNA was caused by PMA in mock-infected cells (Figure 10D), confirming the role of the protein kinase inducer in catecholamine biosynthesis and metabolism under our experimental conditions. These results are in agreement with the ones obtained in respective experiments with HCV virus [33]. On the other hand, PMA did not influence DDC expression levels (Figure 10C,D).

### 3.8. The Catecholamine-Related Cellular Redox Alterations Are Not Related with the Inhibition of DV Replication

**Figure 11:**
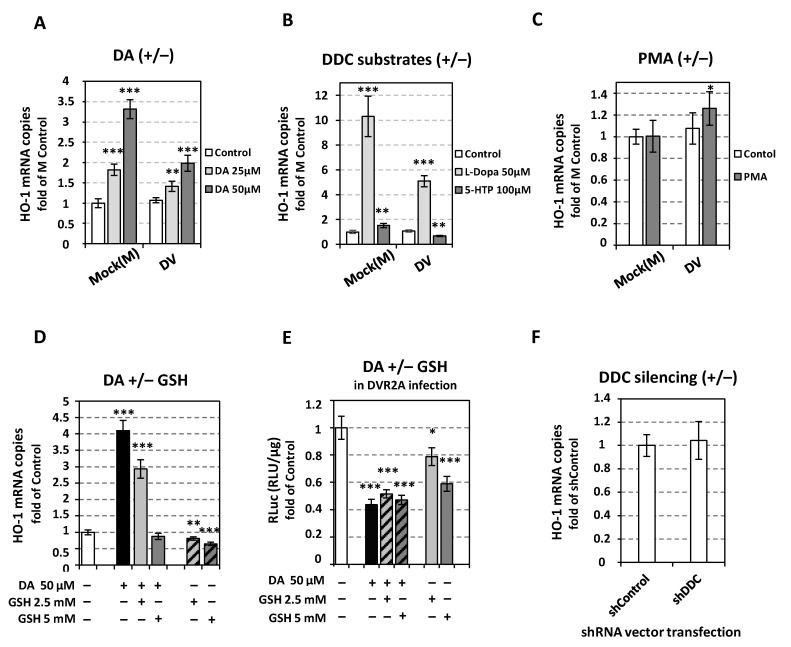
Effect of catecholamine biosynthesis and metabolism pathway on HO-1 mRNA levels, a cellular redox homeostasis marker. (**A**–**E**) Huh7 cells were inoculated with DV (**A**–**C**) (MOI = 0.1), or DVR2A (**E**) (MOI = 0.1) or were mock-infected (mock, M) for 4 h, and then treated with the appropriate concentrations of DA (**A**,**D**,**E**), L-Dopa, 5-HTP (**B**), PMA (**C**), GSH (**D**,**E**), or were mock-treated (Control), for 48 h. (**A**–**D**) RT-qPCR analysis was performed to determine the mRNA amounts of HO-1. The mRNA levels of the housekeeping gene YWHAZ were used for normalization. Mock-infected mock-treated cells (M Control) (**A**–**C**) or infected mock-treated (Control, (−)) cell (**D**,**E**) values were set to one. (**E**) Renilla luciferase activity was quantified. Control (−) cell values were set as one. (**F**) RT-qPCR analysis was performed to determine the mRNA levels of HO-1. Lysates from Huh7.5 cells stably expressing shDDC or shControl were used. shControl-expressing cell values were set as one. The mean values ± standard deviations from three independent experiments in triplicate are featured. * *p* < 0.05, ** *p* < 0.01, *** *p* < 0.001 vs. M Control for (**A**–**C**) or Control (−) for (**D**,**E**).

We have previously shown that catecholamines exogenously applied in human hepatoma cells induce ROS formation and activate the expression of antioxidant response genes [33] in agreement with other reports [12,30]. To verify that catecholamines affect the cellular redox homeostasis under the present experimental conditions, we measured the expression levels of *HO-1*, known to be induced by ROS production, in cells treated with DA and NE, as well as in cells treated with DDC substrates, L-Dopa and 5-HTP. As shown in both mock- and DV-infected cells, the application of catecholamines or of DDC substrates strongly induced HO-1 mRNA levels in a dose-dependent manner (Figure 11A,B and Figure S7C). Treatment with PMA, even though would be expected to enhance the accumulation of catecholamines, through upregulation of TH and DDC [12,82], did not alter the mRNA levels of HO-1 (Figure 11C). This possibly occurs due to its strong positive effect on *MAO-B* expression (Figure 10D) which leads to a concomitant increase in the enzymatic degradation of catecholamines. Upon DV infection the catecholamine-mediated induction of *HO-1* expression was significantly lower (Figure 11A,B). This is in agreement with previous reports supporting that DV enhances the degradation of Nrf2 and as a result downregulates *HO-1* facilitating elevated ROS levels inside the cell [85]. The latter condition favors DV replication in Huh7 cells, as shown by the positive effect of externally applied H_2_O_2_ or by the incubation of infected cells under hypoxic conditions [86], as well as by the negative impact of externally applied reduced glutathione [86,87].

Thus, while on the one hand catecholamines negatively affected DV replication in hepatoma cells, on the other hand these molecules increased ROS levels, as deduced by the upregulated *HO-1* expression, a condition that has been shown to favor DV replication. This suggests that catecholamines affect DV through a different mechanism unrelated to redox status of the cell. In support of this, reduced glutathione (GSH) although lowered the DA-mediated HO-1 mRNA levels (Figure 11D) failed to differentiate the DA-effect on DV replication (Figure 11E). Furthermore, although DV replication was enhanced in shDDC expressing cells, in which DA endogenous production is downregulated, these cells support similar expression levels of *HO-1* compared to shControl-expressing cells (Figure 11F).

### 3.9. DV Suppresses the Expression of the Catecholamine Biosynthetic/Metabolic Pathway Enzymes

**Figure 12:**
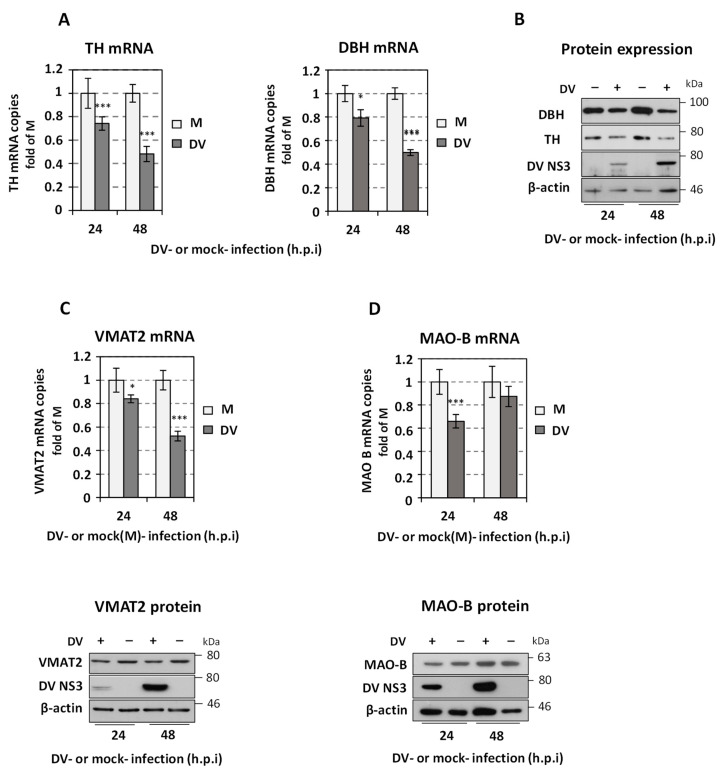
Effects of DV infection on the catecholamine pathway genes expression, except for DDC. After their inoculation with DV (MOI = 1), or mock infection (mock, M), Huh 7 cells were cultured for the displayed h.p.i. ((**A**,**C**,**D**) upper panels) TH, DBH, VMAT2, and MAO-B mRNA levels were determined by RT-qPCR analysis and normalized to YWHAZ mRNA. Values are expressed relative to the ones derived from mock-infected (M) cells, at each time point. The mean values ± standard deviations from three biologically independent experiments in triplicate are featured. * *p* < 0.05, *** *p* < 0.001 vs. mock-infected cells. ((**B**–**D**) lower panels) SDS-PAGE and immunoblot analysis were carried out in lysates of DV-infected (+) and mock-infected (−) cells with the use of antibodies detecting the proteins TH or DBH (**B**), VMAT2 (**C**), MAO-B (**D**) and β-actin (loading control). Anti-NS3 antibody was used to verify viral infection. An experiment that is representative of three independent repetitions is shown.

Then, we investigated whether DV infection affects the expression of other catecholamine biosynthetic and metabolic pathway enzymes, except for DDC. We observed that infection of Huh7 cells with DV at a MOI of 1 downregulated *TH* expression (Figure 12A), which encodes a rate-limiting enzyme of the catecholamine biosynthesis as it produces the precursor L-Dopa. Additionally, we detected that DV had a significant negative impact in the mRNA and protein levels of DBH (Figure 12A,B), which functions downstream of DDC. As the above-mentioned results have shown that suppression of DBH function does not influence DV (Figure 7A), the impact of DV infection on DBH might derive from the lower levels of the upstream biosynthetic enzyme DDC due to DV infection. Indeed, when DDC protein levels are downregulated under conditions not related to a viral infection, such as upon *DDC* silencing, a concomitant reduction of DBH expression occurs [33]. Similarly, viral infection affects the expression of VMAT2 as is evident from the decreased levels of the transporter mRNA and protein (Figure 12C). The latter finding is consistent with the higher extracellular dopamine levels observed in cells infected with a low MOI (Figure 6E), which do not significantly affect cell viability (Figure S3F), as compared to mock-infected cells. These are equivalent to the dopamine levels in plain culture medium not exposed to cells (Medium, Figure 6E), suggesting a suppression of dopamine uptake upon viral infection. Finally, MAO-B mRNA amounts were diminished, especially at 24 h.p.i, upon DV infection, but protein levels were not significantly influenced (Figure 12D).

### 3.10. Catecholamine Biosynthesis/Metabolism Negatively Affects DV Replication in Other Cell Lines

**Figure 13:**
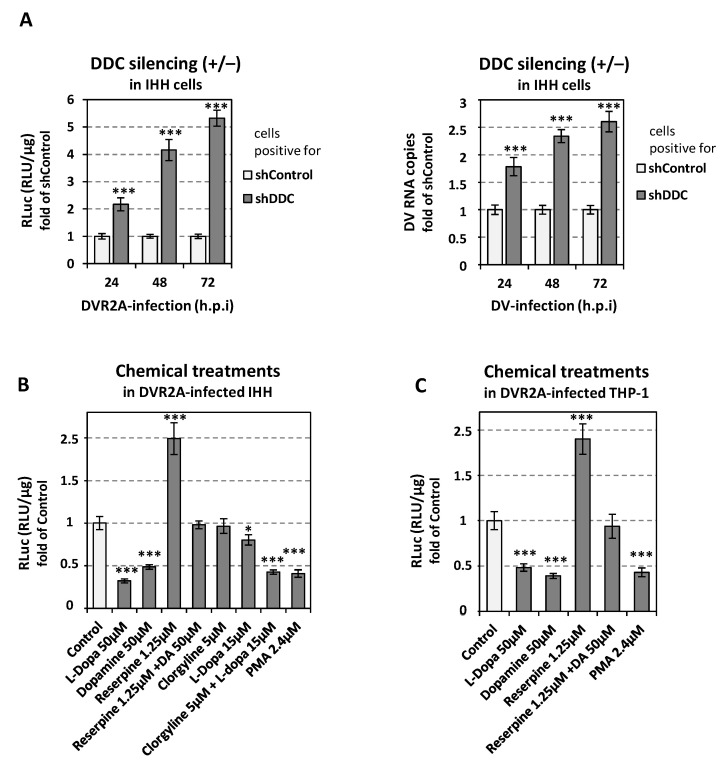
The activity of the catecholamine biosynthesis/metabolism pathway negatively regulates DV replication in immortalized human hepatic (IHH) and blood monocytic (THP-1) cell lines. (**A**) 24 h after electroporation of IHH cells with the shDDC vector, or the shControl vector, cells were infected by DVR2A reporter virus (MOI = 0.1) ((**A**)-Left) or DV (DV-2 16681 strain, at an MOI = 0.1) ((**A**)-Right) for 4 h, and after virus inoculum removal were further cultured for the indicated hours post-infection (h.p.i). ((**A**)-Left) Levels of Renilla luciferase activity (RLuc)*,* indicative of DV replication, were expressed as RLU/μg of total protein amount. ((**A**)-Right) RT-qPCR analysis was performed to determine DV plus-strand RNA levels. At each time point, values derived from shControl-expressing cells were set to one. *** *p* < 0.001 vs. shControl at each timepoint. (**B**,**C**) After a 4 h inoculation of IHH (**B**) or THP-1 (**C**) cells with DVR2A (MOI = 0.1) virus, cells were treated with DDC substrates and products, VMAT-specific inhibitor reserpine, MAO-A inhibitor clorgyline and the catecholamine pathway inducer PMA at the indicated concentrations or were mock-treated (Control), and further cultured for 48 h. Then, the cells were lysed and Renilla luciferase activity was determined. Values are compared to the ones of Control cells (Control). Data shown are means ± standard deviations of values from three independent biological experiments in triplicate. * *p* < 0.05, *** *p* < 0.001 vs. Control.

To confirm the bidirectional relationship between catecholamine biosynthesis/metabolism and DV replication in other cell lines, we used the immortalized human hepatic cell line IHH and the hematopoietic lineage cell line THP-1. THP-1 was selected as blood monocytes are among the prime targets of DV in vivo [88]. Firstly, we examined the effect of *DDC* silencing on DVR2A in infected IHH cells. IHH cells were transfected via electroporation with the shDDC vector or with the corresponding control vector (shControl) and 24 h.p.e. the cells were infected with DVR2A (MOI = 0.1). As shown in Figure 13, *DDC* silencing significantly increased viral replication-derived RLuc activity (Figure 13A-Left) and viral RNA levels (Figure 13-Right). To study the effect of the activity of the catecholamine biosynthesis/metabolism pathway on DV replication, in IHH- and THP1- cells, we infected cells with DVR2a for 4 h and then we treated them for 48 h with DDC substrates, DDC products, VMAT-specific inhibitor reserpine, MAO-A inhibitor clorgyline and the catecholamine biosynthesis inducer PMA. L-Dopa, dopamine and PMA treatment decreased virus-derived RLuc activity in both cell lines suggesting that the upregulation of catecholamine biosynthesis downregulates viral replication (Figure 13B,C). Reserpine upregulated the levels of DV-derived luciferase activity when administrated alone, and attenuated the effect of dopamine on the virus in combinatory treatments (Figure 13B,C), in both cell lines. This suggests that VMAT2 inhibition enhances DV replication. Lastly, in DV-infected IHH cells, clorgyline did not seem to alter viral replication when used alone, but increased the negative effect of L-Dopa in combinatory treatments as shown in Figure 13B. These results are in agreement with data obtained from Huh7 hepatic cell line and suggest that catecholamines negatively affect DV replication.

### 3.11. DV Infection Downregulates the Catecholamine Biosynthesis/Metabolism Pathway in Other Cell Lines

**Figure 14:**
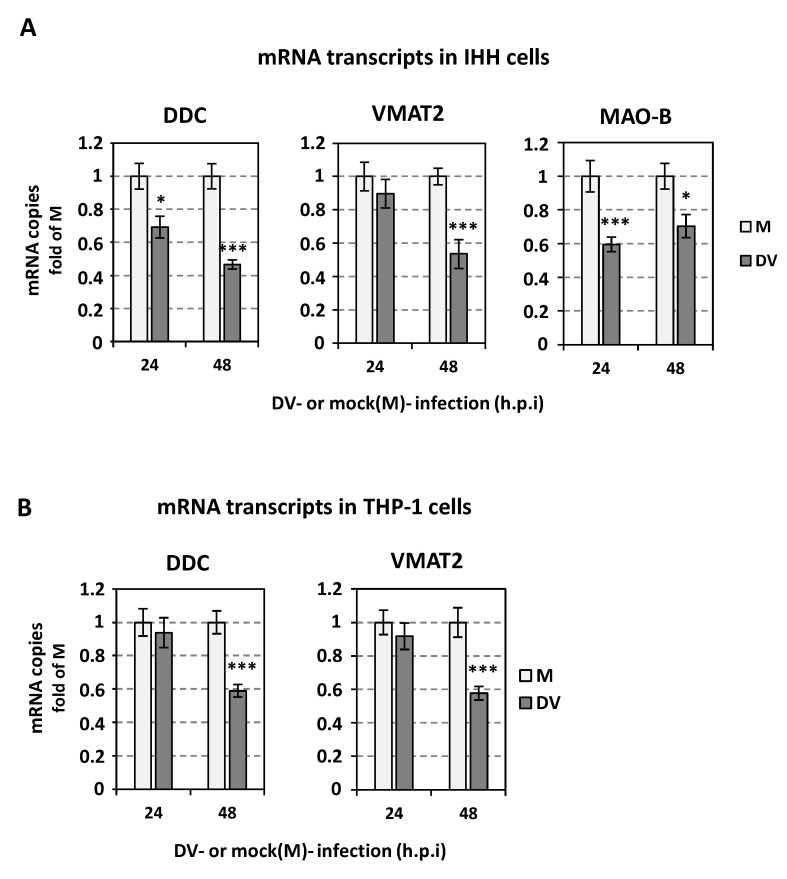
DV infection downregulates the expression of the catecholamine biosynthesis/metabolism related genes in immortalized human hepatic (IHH) and blood monocytic (THP-1) cell line. IHH (**A**) and THP-1 (**B**) cells, were inoculated with DV (MOI = 1), or mock-infected (mock, M) and further cultured for the indicated h.p.i. DDC, VMAT2, and MAO-B mRNA levels were determined by RT-qPCR analysis and normalized to YWHAZ mRNA. Values are expressed relative to the ones derived from mock-infected (mock, M) cells, at each time point. The mean values ± standard deviations from three independent experiments in triplicate are featured. * *p* < 0.05, *** *p* < 0.001 vs. M at each timepoint.

To examine whether DV infection influences the catecholamine biosynthetic/metabolic pathway in IHH and THP-1 cells, we infected cells with DV (MOI = 1) and analyzed the effects on the expression of the related genes. As shown in Figure 14, DV infection downregulated the mRNA levels of DDC, VMAT2 and MAO-B (Figure 14A) in IHH cells, indicating a generalized downregulation of the catecholamine biosynthesis/metabolism pathway. Similarly, DV infection had a negative impact on DDC and VMAT2 mRNA levels (Figure 14B) in THP1 cells, while MAO-B mRNA was not detectable in this monocytic cell line (data not shown).

## 4. Discussion

Here, we outline that DV replication is restricted by the production, uptake and metabolism of catecholamines in liver cells, and, in turn, DV has developed ways to counteract the effect of catecholamines by reducing their cytoplasmic levels.

First, we confirmed the negative effect of DDC on DV in Huh7 cells by performing *DDC* gene silencing and clarified that DDC specifically influences viral genome replication and not viral translation. This is in accordance with our previous data indicating amelioration of viral replication upon treatment with carbidopa, an inhibitor of DDC enzymatic activity [21]. Downregulation of DDC expression was also shown to reverse the loss of cell viability of DV-infected cells observed at later hours p.i., while overexpression of DDC exacerbated the decrease in intracellular ATP levels during viral infection. This agrees with the previously reported function of DDC in cell apoptosis [24] and the implication of PI3K/AKT signaling in DDC-DV association [21,25].

Besides our previous finding that DDC impacts DV replication through its physical and functional interaction with PI3K [21,25], this study indicated that DV replication was downregulated by the intracellular conversion of DDC enzyme substrates into products. Specifically, L-Dopa and 5-HTP negatively affected DV replication, and this effect was lessened significantly by *DDC* silencing. The lack of any alteration in the DDC mRNA levels upon treatment of infected cells with the DDC substrates, confirms that the negative effect of DDC substrates on DV infection is not due to differences in *DDC* expression.

In addition to their biosynthesis, the external administration of DDC enzyme products dopamine (DA) and serotonin (5-HT) in the cells had an adverse impact on DV replication as well. The antiviral activity of DA on DV was not compensated by the use of PCZ, a known D2 dopamine receptor antagonist suggesting that it is not related to the activation of D2 [62,63]. These data combined with the observation that the effect of DA on DV was not accompanied by an alteration in DDC levels, favors a possible role of DA uptake on viral replication. The importance of the catecholamine uptake on DV was directly addressed by the findings derived from the treatment of cells with the VMAT-specific inhibitor reserpine [26,27,28,29,67,68,69,70]. We observed that reserpine positively affected viral replication, while abrogated the dopamine uptake from the cell culture medium. The most pronounced positive effect on DV was apparent in the lowest concentration of reserpine applied. This could be explained by the antioxidant properties of reserpine in higher concentrations (>2.5 μΜ) (Figure S7D), occurring through epigenetic modulation and subsequent activation of Nrf2 pathway [89,90], an unfavorable condition for DV replication [85]; moreover, reserpine decreased *OCT1* transcription. OCT1 is an important cell membrane catecholamine transporter in the liver, capable of importing catecholamines and L-Dopa [72,73,74]. Thus, in addition to the abolishment of the cellular import of dopamine, the blockage of L-Dopa uptake could also contribute to the reserpine-mediated induction of DV replication. Reserpine also weakened the negative role of the applied dopamine on the virus. The finding that DDC mRNA levels remain unaffected in mock-infected cells upon treatment with reserpine, excludes the possibility that the effect of reserpine on DV could be due to a modulation of *DDC* expression.

The lack of a positive effect on DV replication in shDDC expressing cells upon reserpine treatment is possibly related with a more reduced cell environment as indicated by the downregulation of HO-1 mRNA levels in these conditions. The reduced environment, which does not favor DV replication [86,87], is possibly created by the lower intracellular catecholamine levels due to *DDC* silencing and reserpine-mediated inhibition of uptake. This is in agreement with previous reports suggesting that catecholamines is a strong source of ROS for the cell [91,92].

Τhe opposing relation between catecholamine biosynthesis and DV replication was further confirmed by studying the involvement of other biosynthetic (TH, DBH) and metabolic (MAO-A/B) enzymes of the pathway, treating the cells with their products or inhibitors. Indeed, the exogenous application of NE was found to inhibit DV replication; however, the use of the DBH-specific inhibitor nepicastat [76], failed to affect DV, as we have previously shown for HCV [33]. These data, combined with the ones regarding the catecholamine storage inhibitor reserpine, suggest that the uptake and presence of NE in the cell cytosol diminishes DV replication, while this does not possibly happen when NE is restricted inside the storage vesicles. The next enzyme of the pathway following DBH, PNMT which catalyzes the conversion of NE to epinephrine seemed not to be expressed in Huh7 cells. This is in agreement with reports supporting the presence of a nonspecific N-methyltransferase synthesizing epinephrine [14] in the liver tissue.

The role of catecholamines metabolism on DV was examined by performing MAO inhibition, which prevents the enzymatic degradation of monoamines, putatively increasing their cytosolic levels and/or favoring their autooxidation. Interestingly, in contrast to what we have shown with HCV [33], MAO inhibitors had no effect on DV unless their use was combined with L-Dopa or 5-HTP in concentrations that do not influence DV replication. These data suggest that DV is less sensitive than HCV to the elevated concentrations of cytosol-located catecholamines. Similarly, the co-treatment of DV-infected cells with DDC substrates and clorgyline or the VMAT-inhibitor reserpine, resulted in the downregulation of viral replication. In the combined treatment, the DDC-produced dopamine is expected to be accumulated in the cell cytoplasm, implying that when elevated levels of catecholamines are present inside the cell, the available cytoplasmic storage vesicles are insufficient to neutralize the levels of cytosol-located catecholamine. As a result, DV replication is inhibited. MAO-A and -B mRNA levels were reduced upon reserpine treatment in a DV infection-independent manner, verifying a connection between VMAT activity and *MAO-A* and -*B* expression. In contrast, the activity of MAO did not seem to influence DDC, as no impact on DDC expression was observed upon clorgyline treatment, in consistence with previous reports [81,93], similarly to the aforementioned lack of effect of reserpine on DDC; moreover, *DDC* silencing did not affect VMAT2 expression, but reduced MAO-B expression (Figure S2C). Thus, catecholamine synthesis may not influence their uptake/storage and vice versa; however, both the synthesis and uptake/storage of catecholamines regulate the rate of catecholamine enzymatic oxidation process.

Forskolin and PMA, that induce catecholamine biosynthesis through PKA and PKC activation, negatively affected DV replication, as in the case of HCV [33]. Τhe induction of catecholamine pathway in the presence of PMA was confirmed by the increased mRNA levels of TH and MAO-B. The lack of any PMA-mediated alteration in *DDC* expression is in accordance with previous cell culture data [94]; moreover, PMA could also act directly on DV NS5 impeding viral replication [95].

As the catecholamine biosynthesis/metabolism exerted a negative effect on DV replication, we examined whether DV can affect the different steps of the pathway, apart from the already known virus effect on DDC. Actually, DV infection strongly downregulated the expression of TH, DBH and VMAT2, while transient negative effects were observed also concerning the level of MAO-B mRNA. The latter could be a result of the lower levels of the cytosol-located catecholamines due to virus-mediated effects on their synthesis (TH, DBH) and uptake (VMAT2). The elimination of DV-mediated suppression on *MAO-B* expression at the later time points p.i. could be related to the need of the virus to compensate for the increased levels of catecholamine autooxidation that occur as a result of the infection-caused oxidative environment at late hours [96]. Interestingly, the expression of TH, DDC and VMAT2, the products of which have been shown to form a functional complex in the cell [97], is similarly affected by DV infection. Thus, the virus could possibly succeed in the downregulation of these genes by targeting a common factor that controls their mRNA levels. Indeed, an ankyrin repeat protein designated V-1 has been suggested to act as a possible coordinate regulator of the expression of TH, DDC, DBH and PNMT mRNAs [75]; moreover, consistently with the downregulation of *VMAT2* expression, we observed a suppression of dopamine uptake from the cell culture medium upon viral infection. The negative DV-mediated effect on DDC [21] was observed not only after viral infection, but also in the context of viral subgenomic replicon. This effect is sustained even for the non-replicative sgDVR2A-GND RNA, at later than 8 h post-transfection, which indicates that at least the viral RNA, or the expression of viral non-structural proteins, has a critical role in the regulation of *DDC* expression (Figure S9).

Interestingly, apart from the data obtained for the hepatic cell line (Huh7), we observed similar results for the relationship between DV and catecholamine biosynthesis/metabolism in two other cell lines, the immortalized human hepatic cell line (IHH) and the monocytic cell line THP-1.

The relationship between catecholamine biosynthesis/metabolism and viral replication is not restricted to *Flaviviridae* family [33,86] as the pathogenesis of several viruses has been related to this pathway [34,35,36,37,38,39,40,41,42,43,44]. Our previous work on SARS-CoV-2, another single-stranded RNA virus, has revealed a significant correlation between DDC expression and viral infection in both nasopharyngeal swab samples of COVID-19 patients and infected cultured airway epithelial cells [34]. SARS-CoV-2 was suggested to suppress DDC to favor its propagation. On the other hand, no association with the catecholamine pathway was observed for other viruses causing respiratory inflammation such as influenza A and B.

Previously, we have reported that elevated levels of ROS, produced during hypoxic reprogramming of cell metabolism, act in favor of DV replication [86]. This was confirmed by the positive effect of externally applied H_2_O_2_ [86] and the negative impact of reduced glutathione on DV infection [86,87], as well as the opposite effect that these treatments had on the expression of antioxidant response (ARE) genes. The supply of cells with catecholamines or L-Dopa was found to induce the expression of ARE-genes, in agreement with previous studies of our group and others [12,30,33]; however, under these conditions, an inhibitory effect on DV replication was observed. This suggests that catecholamines negatively affect DV replication with an up-to-date unknown mechanism, the effects of which cannot be nullified by the expected positive effect of the catecholamines-mediated ROS production. The existence of such a mechanism was supported by the following data: *DDC* silencing, although did not affect *HO-1* expression, resulted in a strong enhancement of DV replication. The concurrent administration of DA and GSH did not enlarge the negative impact of DA on DV, although GSH fully reverted the DA-mediated *HO-1* induction. Interestingly, in HCV-infected cells, GSH reverts the downregulation of viral replication caused by DA treatment [33]. Thus, this mechanism appears to be specific for DV [86,96], as in the case of HCV the replication of which is sensitive to catecholamine-mediated ROS production [98,99] the use of GSH restores both the redox status of the cell and the HCV replication levels.

Additionally, PMA, although induced catecholamine biosynthesis inhibiting DV replication, did not alter *HO-1* expression. The latter may be related with the PMA-exerted strong enhancement of *MAO* expression that is expected to lessen catecholamines autooxidation, lowering the subsequent ROS production [12,30,100].

Upon catecholamine depletion, as in the case of reserpine-treated DV-infected shDDC expressing cells, where both the biosynthesis and uptake of catecholamines are blocked, the catecholamine-related mechanism that exerts effects on DV replication is downregulated; however, under these conditions, ROS levels are reduced as shown by the lower levels of *HO-1* expression, and this could account for the lack of an effect of reserpine treatment in these cells.

Moreover, the catecholamine-mediated effect on DV replication was not linked with upregulation of cell death, since concentrations of dopamine or reserpine that had no effect on cellular viability (Figure S3A,D) and exhibited no effect on the levels of p-AKT protein (Figure S10A,B), differentially affected viral replication. Additionally, the cell bioenergetics seems not to be involved in the inhibition of DV replication by the catecholamines, since both *DDC* silencing, and NE-treatment upregulated the intracellular levels of ATP but resulted in opposite effects on DV replication.

Thus, apart from the previously reported interaction of DDC with PI3K that mediates DDC-DV association [21,25], other mechanisms exerted from the enzymatic products of the pathway (DA, NE) can also contribute to the relationship between the catecholamine pathway and DV infection. For example, regulation of gene expression by DA and serotonin through post-translational modifications of histones in a process called dopaminylation [101] and serotonylation [102] respectively, may be implicated; moreover, the oxidized metabolites of catecholamines, known as quinones, could possibly be responsible for DV replication suppression. Indeed, anti-dengue virus activities have been attributed to quinone derivatives [103]. Interestingly, the co-treatment with clorgyline and reserpine that is expected to trap catecholamines in the cell cytosol and elevate the quinone levels, attenuate the enhancement of DV replication produced by reserpine treatment alone. Finally, NE has been shown to induce an inflammatory response [104], which may be related with the observed DV suppression.

The results of the present study highlighted a complex bidirectional relationship between DV and important components of the catecholamine biosynthetic and metabolic pathway, elucidating novel determinants of viral replication and reinforcing published data on the function of bioactive amines in the periphery.

## Figures and Tables

**Table 1:** Priming oligonucleotides used for RT-qPCR analysis.

Target	Orientation	Sequence (5′-3′)
*DV-S*	Forward	GAAAGACCAGAGATCCTGCTGTCT
*DV-A*	Reverse	ACCATTCCATTTTCTGGCGTT
*DDC*	Forward	GAACAGACTTAACGGGAGCCTTT
	Reverse	AATGCCGGTAGTCAGTGATAAGC
*TH*	Forward	GGAAGGCCGTGCTAAACCT
	Reverse	GGATTTTGGCTTCAAACGTCTC
*DBH*	Forward	GCCTTCATCCTCACTGGCTACT
	Reverse	CAGCACTGTGACCACCTTTCTC
*MAO-A*	Forward	GGGCTGCTACACGGCCTACT
	Reverse	GACCTCCCTAGCTGCTCGTTCT
*MAO-B*	Forward	GGAGCCAGTGCATTATGAAGA
	Reverse	GCCTGCAAAGTAAATCCTGTC
*VMAT2*	Forward	CGGATGTGGCATTTTGTATGG
	Reverse	TTCTTCTTTGGCAGGTGGACTTC
*OCT1*	Forward	CACCCCCTTCATAGTCTTCAG
	Reverse	GCCCAACACCGCAAACAAAAT
*HO-1*	Forward	ATGACACCAAGGACCAGAGC
	Reverse	GTGTAAGGACCCATCGGAGA
*YWHAZ*	Forward	GCTGGTGATGACAAGAAAGG
	Reverse	GGATGTGTTGGTTGCATTTCCT

## Data Availability

All relevant data are within the manuscript and its Supporting Materials.

## Supplementary Materials

The following supporting information can be downloaded at: https://www.mdpi.com/article/10.3390/v14030564/s1, Supplementary Materials and Methods, Figure S1: Kinetics of virus replication, Figure S2: Downregulation of DDC, VMAT and MAO-B expression upon DDC silencing. (A-left panel), Figure S3: Effect of catecholamine pathway-associated enzyme substrates and products, as well biosynthetic enzymes and transporters inhibitors or inducers on intracellular ATP levels at three different cell lines, Figure S4: Expression of DDC in response to DA, reserpine, nepicastat, NE and clorgyline treatment, Figure S5: Effect of DA and reserpine on DBH mRNA levels, Figure S6: Effect of NE, as well VMAT and MAO inhibitors on DV replication, Figure S7: (A): Effect of the DV infection on the expression of OCT-1, (B) Reserpine increases the sensitivity of DV replication in 5-HTP, (C) Effect of catecholamine biosynthesis and metabolism pathway on HO-1 mRNA levels, (D) Reserpine diminishes the negative effect of H_2_O_2_ on cell viability, Figure S8: DBH inhibition (nepicastat) has no effect on DV replication, Figure S9: Effect of DV genome translation and replication on DDC mRNA levels, Figure S10: Effect of DA and reserpine on the levels of p-Akt protein.
