# Supplementary material for: Dengue Virus Replication Is Associated with Catecholamine Biosynthesis and Metabolism in Hepatocytes
Source: Viruses. 2022 Mar 9;14(3):564. doi: 10.3390/v14030564 (PMC8948859; doi:10.3390/v14030564)
Supplement: Supplementary file 1 [file viruses-14-00564-s001.zip › viruses-1557112-supplementary.pdf]

viruses

Supplementary Data for

Dengue Virus Replication is associated with Catecholamine Biosynthesis and Metabolism in hepatocytes

George Mpekoulis [1†], Vassilina Tsopela [1†], Anna Chalari [1†], Katerina I. Kalliampakou [1], Georgios Panos [1], Efseveia Frakolaki [1‡], Raphaela S. Milona [1], Diamantis C. Sideris [2], Dido Vassilacopoulou [2] and Niki Vassilaki [1,*]

[1] Laboratory of Molecular Virology, Hellenic Pasteur Institute (HPI), Athens, Greece
[2] 1st Department of Critical Care Medicine & Pulmonary Services, GP Livanos and M Simou Laboratories, Evangelismos Hospital, Athens Medical School, National & Kapodistrian University of Athens, Athens, Greece
† Equal contribution
‡ Deceased
* Correspondence: nikiv@pasteur.gr; Tel.: +30-210-647-8875

Citation: Mpekoulis, G.; Tsopela, V.; Chalari, A.; Kalliampakou, K.I.; Panos, G.; Frakolaki, E.; Milona, R.S.; Sideris, D.C.; Vassilacopoulou, D.; Vassilaki, N. Dengue Virus Replication is associated with Catecholamine Biosynthesis and Metabolism in hepatocytes. *Viruses* **2022**, *14*, 564. https://doi.org/10.3390/v14030564

Academic Editors: Ioannis Karakasiliotis, Apostolos Beloukas and Serafeim Chaintoutis

Received: 10 January 2022
Accepted: 3 March 2022
Published: 9 March 2022

Publisher's Note: MDPI stays neutral with regard to jurisdictional claims in published maps and institutional affiliations.

Copyright: © 2022 by the authors. Submitted for possible open access publication under the terms and conditions of the Creative Commons Attribution (CC BY) license (https://creativecommons.org/licenses/by/4.0/).

1. Supplementary Materials and Methods

1.1. Antibodies and Chemicals

Hydrogen peroxide (H_2O_2) was purchased by Sigma-Aldrich (Taufkirchen, Germany). P-Akt1/2/3 mouse monoclonal antibody (clone B-5; Santa Cruz Biotechnology) was used at a dilution of 1:500.

2. Supplementary Figures

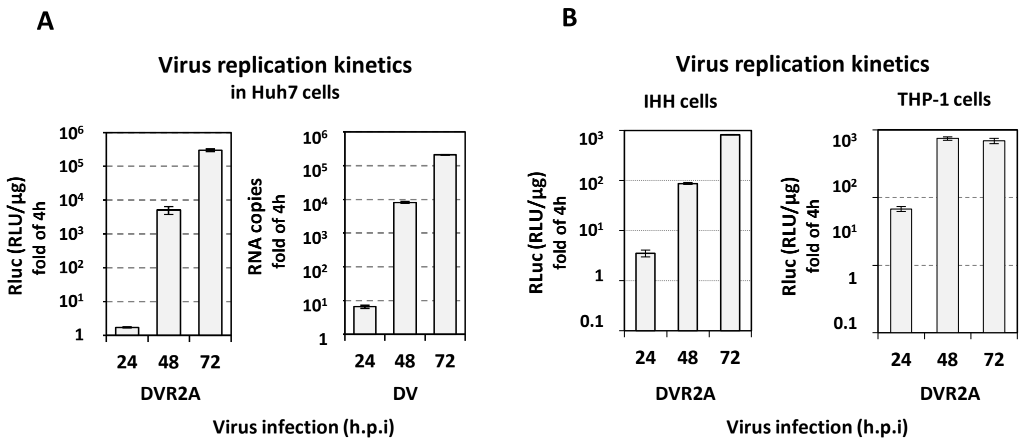

Figure S1. Kinetics of virus replication. Twenty four hours after electroporation of Huh7 (A) IHH (B) or THP-1 (C) cells with shRNA control vector (shControl), cells were infected with (A-Left,B,C) DVR2A reporter virus (at an MOI=0.1) or (A-Right) DV (DV-2 16681 strain, at an MOI = 0.1) and lysed at 4, 24, 48, and 72 h.p.i. (**A-Left,B**) Levels of Renilla luciferase (RLuc) activity, indicative of DV replication, were expressed as RLU/μg of total protein amount. (**A-Right**) RT-qPCR analysis was performed to determine DV plus-strand RNA levels. Values are expressed relatively to the ones obtained at 4 h. The mean values ± standard deviations from three biological independent experiments in three technical replicates are presented.

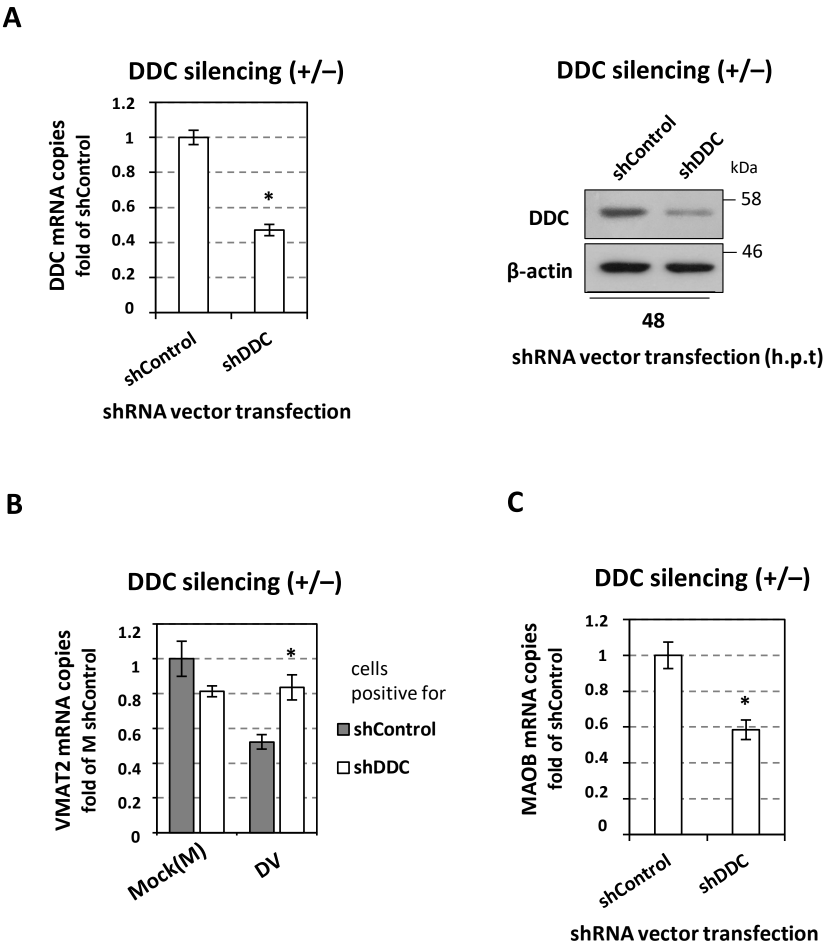

Figure S2. Downregulation of DDC, VMAT and MAO-B expression upon DDC silencing. (**A-Left**) RT-qPCR in Huh7.5 cells stably expressing shDDC vector, that expresses an shRNA targeting DDC mRNA, or the shControl vector was used to quantify DDC mRNA levels that were subsequently normalized toYWHAZ mRNA levels. shControl cell values were expressed as one. The mean values ± standard deviations from three independent experiments in triplicate are presented. *p < 0.001 vs. shControl. (**A-Right**) SDS-PAGE and immunoblot analysis were carried out in lysates of Huh7.5 cells stably expressing shDDC or shControl, by the use of antbodies against the proteins-DDC or β-actin (loading control). (**B**) Huh7.5 cells stably expressing shDDC or shControl were inoculated with DV (MOI = 1) or were mock-infected (mock, M) for 4h, and then cultured for 48 h.p.i. VMAT2 mRNA levels were determined by RT-qPCR and normalized to the ones of housekeeping gene(YWHAZ). Values from M shControl cells were set as one. The mean values ± standard deviations from three independent experiments in triplicate are presented. *p < 0.001 vs. M shControl. (**C**) Quantification of MAO-B mRNA levels was performed by RT-qPCR in Huh7.5 cells stably expressing shDDC or shControl. shControl cell values were expressed as one. *p < 0.001 vs. shControl.

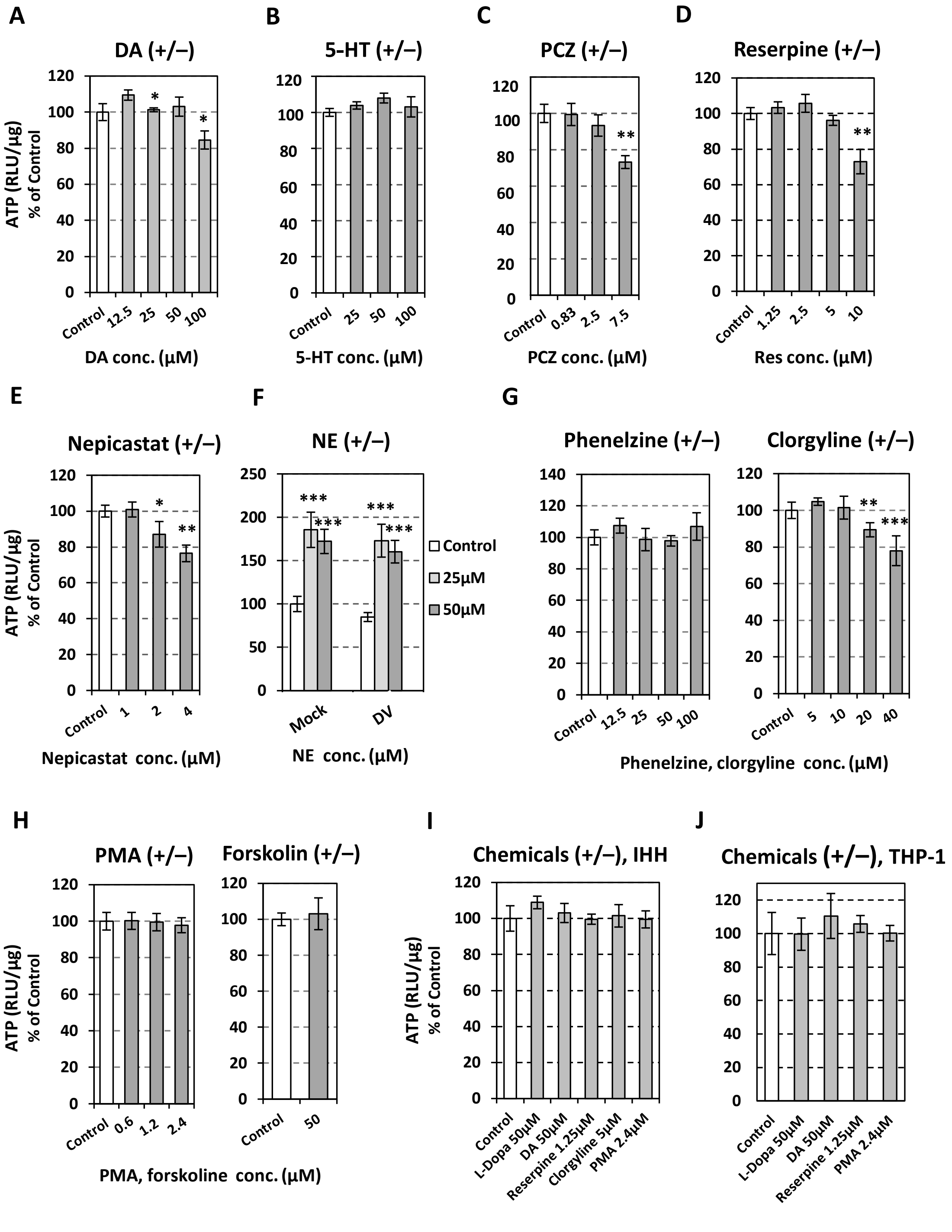

Figure S3. Effect of catecholamine pathway-associated enzyme substrates and products, as well biosynthetic enzymes and transporters inhibitors or inducers on intracellular ATP levels at three different cell lines, Huh7 cells (A-H), IHH (**I**) or THP1(**J**). Huh7 cells were treated with the displayed concentrations of (**A**) DA, (**B**) 5-HT, (**C**) PCZ, (**D**) reserpine, (**E**) nepicastat, (**G**) phenelzine, clorgyline, (**H**) PMA, forskoline or were mock-treated (Control) for 48 h. (**F**) After a 4h infection of Huh7 cells with DV (MOI = 0.1) or mock-infection (M), cells were further treated for 48 h with the pointed concentrations of NE. By the use of a chemiluminescence-based assay, the intracellular ATP was quantified. Control cell values were expressed as one. The mean values ± standard deviations from three independent experiments in triplicate are presented. *p<0.05, **p<0.01, ***p<0.001 vs Control, or vs mock-infected (M) Control.

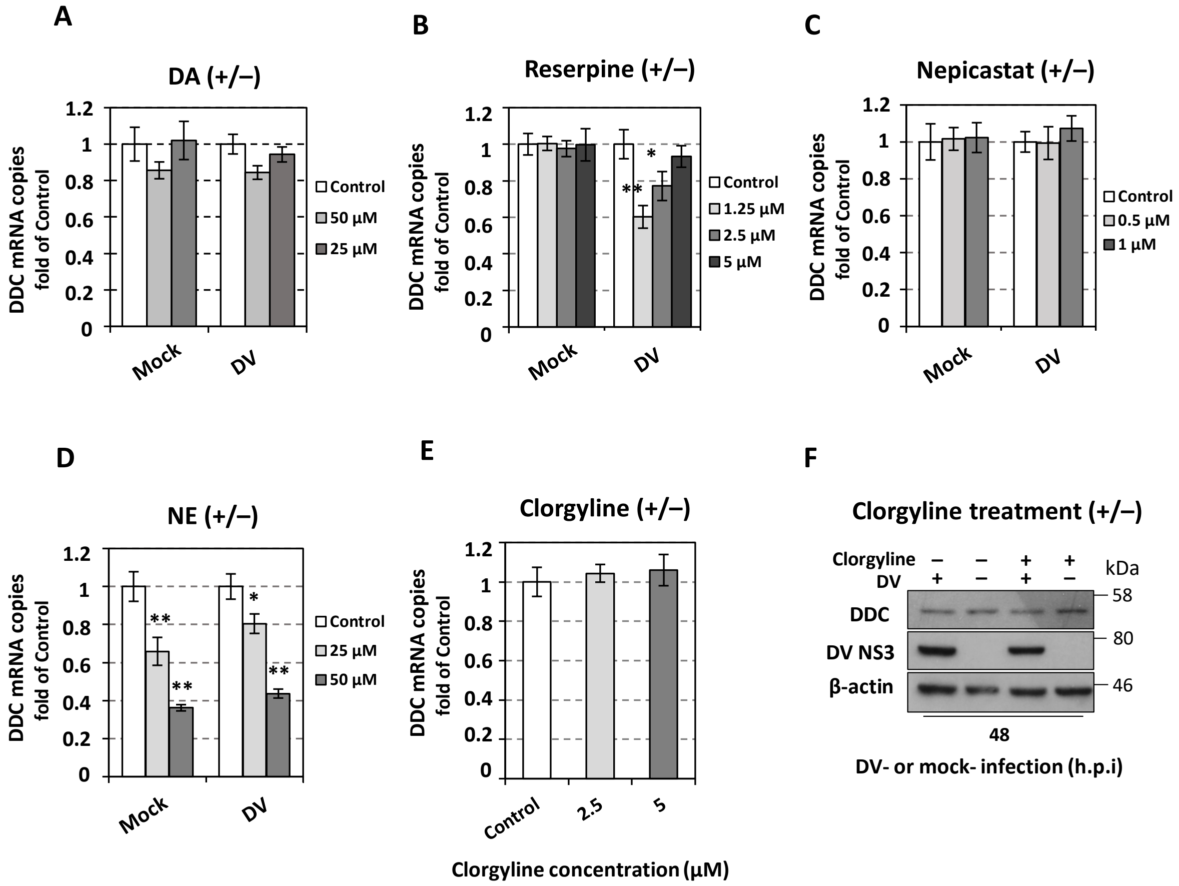

Figure S4. Expression of DDC in response to DA, reserpine, nepicastat, NE and clorgyline treatment. After a 4h inoculation of Huh7 cells with DV (A-D,F) (MOI=0.1) or not (mock), cells were subsequently treated with different concentrations of (A) DA, (B) reserpine, (C) nepicastat, (D) NE, (E-F) clorgyline or were mock-treated (Control) for 48 h. (**A-E**) RT-qPCR was used for the quantification of DDC mRNA levels that were subsequently normalized to the ones of YWHAZ. Control cell values were set as one. Data shown are means ± standard deviations of values from three independent experiments in triplicate. *$p < 0.01$,**$p < 0.001$ vs. Cont(**F**) S rol. DS-PAGE and immunoblot analysis were carried out in cell lysates of DV (+) or mock-infected(−), treated (+) with 5 μM clorgyline or mock-treated (−) for 48 h, with the use of antibodies against DDC, DV NS3 and β-actin proteins. β-actin served the purpose of loading control. An experiment that is representative of three independent biological repetitions is shown.

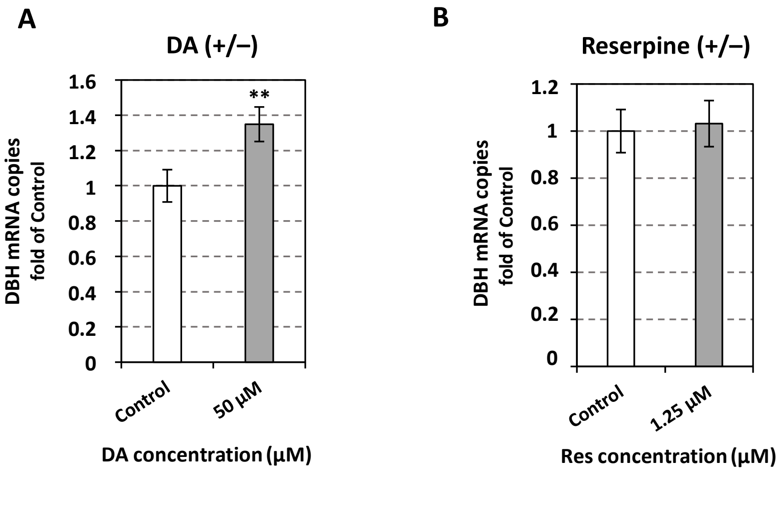

Figure S5. Effect of DA and reserpine on DBH mRNA levels. Huh7 cells were treated with the pointed concentrations of (**A**) DA or (**B**) reserpine, or were mock-treated (Control) and then cultured for 48h. Qantification of DBH mRNA was performed by RT-qPCR and their levels were normalized to the ones of housekeeping gene (YWHAZ). Control cell values were set as one. The mean values ± standard deviations from three independent experiments in triplicate are presented. **p < 0.01 vs. Control.

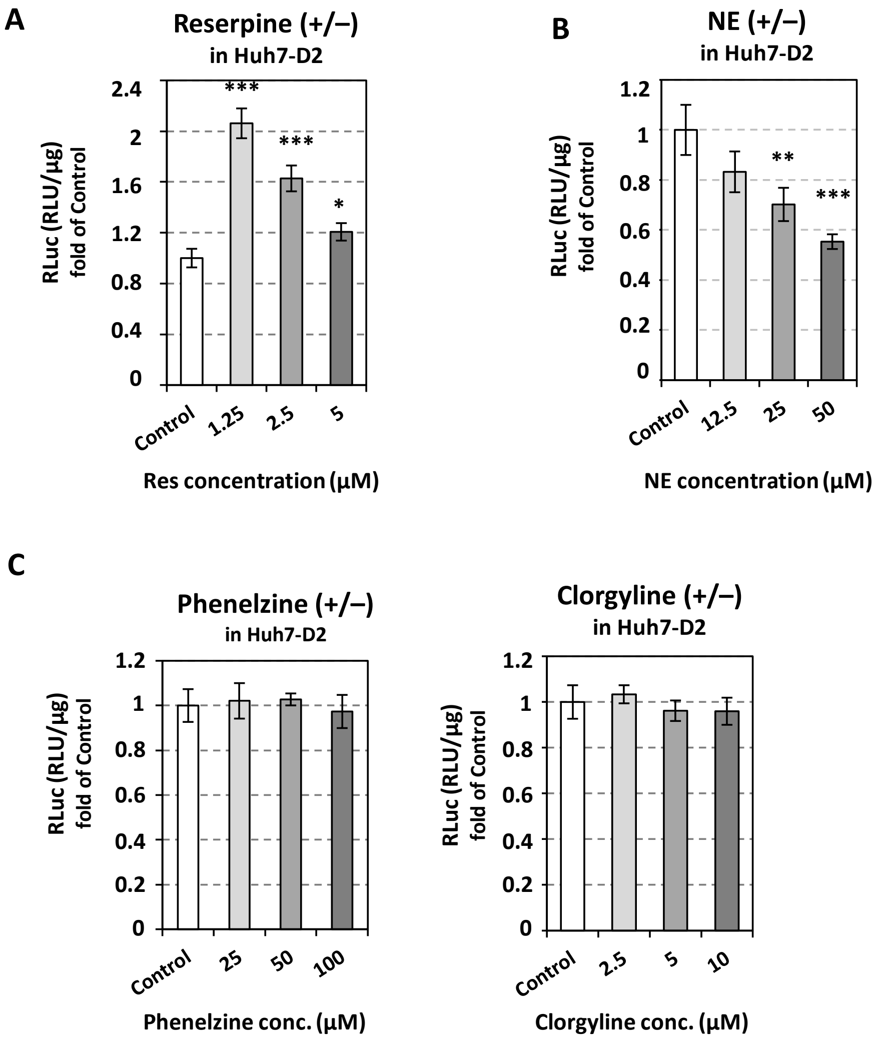

Figure S6. Effect of NE, as well VMAT and MAO inhibitors on DV replication. Huh7-D2 stable cell line that harbors the DV subgenomic replicon was treated with different concentrations of (**A**) reserpine, (**B**) NE, (**C**) phenelzine, clorgyline, or was mock-treated (Control) for 48h. Levels of Renilla luciferase activity (RLuc), indicative of DV replication, were expressed as RLU/μg of total protein amount. Control cell values were set as one. Data shown are means ± standard deviations of values from three independent experiments in triplicate.*p < 0.05, **p < 0.01, ***p < 0.001 vs. Control.

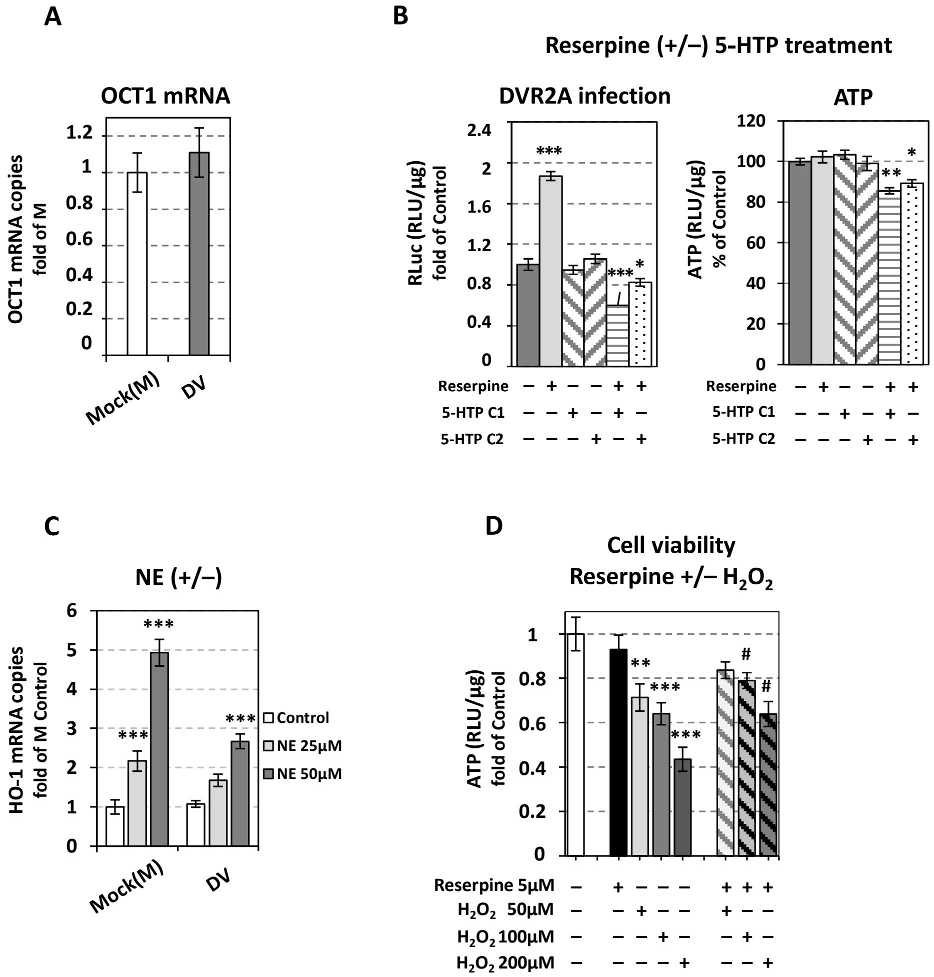

Figure S7. (**A**) Effect of the DV infection on the expression of OCT-1. Huh7 cells were inoculated with DV (MOI = 1), or were mock-infected (mock, M) and further cultured for 48h. RT-qPCR analysis of OCT-1 mRNA levels was performed and normalized to YWHAZ mRNA. Values are expressed relative to the ones derived from mock-infected (M) cells. (**B**) Reserpine increases the sensitivity of DV replication in 5-HTP. After a 4 h inoculation of Huh7 cells with DVR2A (MOI = 0.1), cells were treated with reserpine 1.25 μM in the presence or not of 5-HTP or were mock-treated (Control, (−)), for 48 h. (**B-Left**) Cells were then lysed and RLuc was determined. Control cell values were set as one. (**B-Right**): Intracellular ATP levels were determined. Control cell values were set as 100%. (**C**) Effect of catecholamine biosynthesis and metabolism pathway on HO-1 mRNA levels. Huh7 cells were inoculated with DV (MOI=1) or were mock-infected (mock, M) for 4 h, and then treated with the appropriate concentrations of NE or were mock-treated (Control), for 48 h. RT-qPCR analysis was performed to determine the mRNA amounts of HO-1. Mock-infected mock-treated cells (M Control) values were set to one. (**D**) Reserpine diminishes the negative effect of H_2O_2 on cell viability. Huh7.5 cells were treated (+) with the pointed concentration of reserpine, in the presence of various concentrations of H_2O_2, or were mock-treated (Control, (−)) for 48 h totally, and medium containing reserpine and H_2O_2 was renewed 4 h before the lysis. By the use of a chemiluminescence-based assay, the intracellular ATP was quantified. Control cell values were expressed as one. The mean values ± standard deviations from three independent experiments in triplicate are presented. *$p<0.05$,**$p<0.01$, ***$p<0.001$ vs Control (−).

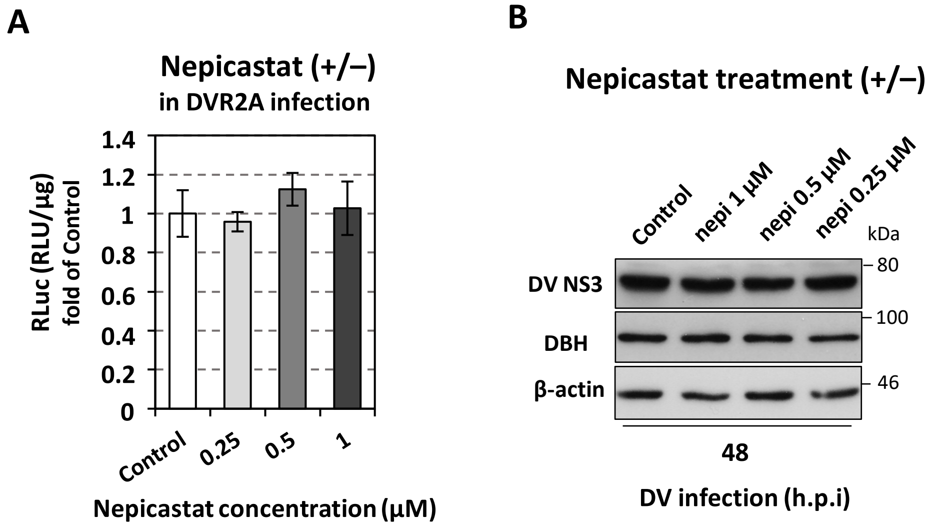

Figure S8. DBH inhibition (nepicastat) has no effect on DV replication. After a 4 h inoculation of Huh7 cells with the reporter DVR2A (MOI = 0.1) (A) or non-reporter DV (MOI = 0.1) (B) and subsequent inoculum removal, cells were treated with different concentrations of nepicastat or were mock-treated (Control) for 48 h. (**A**) RLuc activity, indicative of DV replication, was determined. Control cell values were set as one. The mean values ± standard deviations from three independent experiments in triplicate are featured. (**B**) SDS-PAGE and immunoblot analysis were carried out in lysates of cells infected by DV and subsequently treated with nepicastat for 48h. Antibodies detecting DV NS3, DBH or β-actin (loading control) proteins were used. An experiment that is representative of three independent repetitions is presented.

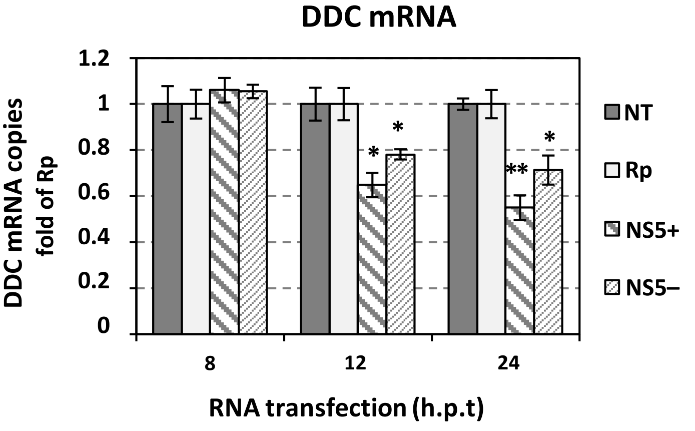

Figure S9. Effect of DV genome translation and replication on DDC mRNA levels. Huh7 cells were electroporated or not (NT, not transfected) with in vitro produced sgDVR2A (NS5+) or its replication defective mutant, sgDVR2A-GND (NS5-) (10 µg RNA/4 × 106 cells), and then cultured for the displayed h p.e. Cells electroporated with a capped-polyadenylated Renilla luciferase expressing RNA (Rp) were considered as control. RT-qPCR analysis was performed to determine the mRNA amounts of DDC and the mRNA of the housekeeping gene (YWHAZ) was used for normalization. Values are expressed relative to the ones derived from (Rp) cells at each time-point. Data shown are means ± standard deviations of values from three independent experiments in triplicate. *p < 0.01, **p < 0.001 vs. Rp.

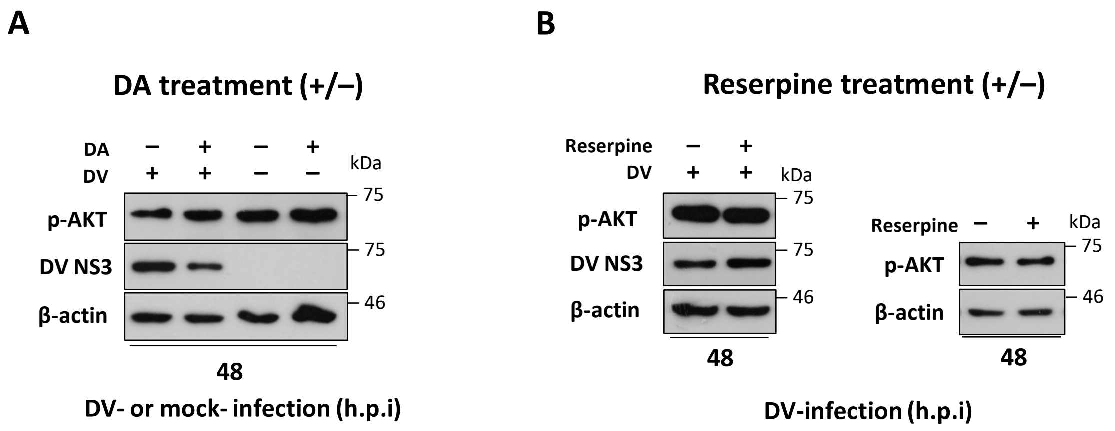

Figure S10. Effect of DA and reserpine on the levels of p-Akt protein. After a 4h infection of Huh7 cells with DVR2A (MOI = 0.1) virus (+) or mock-infection(−), cells were subsequently treated (+) for 48h with (**A**) DA (50 μM), (**B**) reserpine (1.25 μM) or were mock-treated (Control, (−)). SDS-PAGE and immunoblot analysis were carried out in lysates using antibodies against the proteins p-Akt, DV NS3 and β-actin (loading control). An experiment that is representative of three independent repetitions is shown.
